# *Halostachys caspica* pathogenesis-related protein 10 acts as a cytokinin reservoir to regulate plant growth and development

**DOI:** 10.3389/fpls.2023.1116985

**Published:** 2023-04-26

**Authors:** Yudan Feng, Yanpeng Ren, Hua Zhang, Youqiang Heng, Zhanxin Wang, Yan Wang

**Affiliations:** ^1^ Xinjiang Key Laboratory of Biological Resources and Genetic Engineering, College of Life Science and Technology, Xinjiang University, Urumqi, China; ^2^ Key Laboratory of Cell Proliferation and Regulation Biology of Ministry of Education, College of Life Sciences, Beijing Normal University, Beijing, China

**Keywords:** pathogenesis related protein 10, growth and development, cytokinin, crystal structure, ligand interactions

## Abstract

Pathogenesis-related class 10 (PR-10) proteins play a role in plant growth and development, but the underlying molecular mechanisms are unclear. Here, we isolated a salt-induced *PR-10* gene from the halophyte *Halostachys caspica* and named it *HcPR10*. *HcPR10* was constitutively expressed during development and HcPR10 localized to the nucleus and cytoplasm. HcPR10-mediated phenotypes including bolting, earlier flowering, increased branch number and siliques per plant are highly correlated with increased cytokinin levels in transgenic Arabidopsis. Meanwhile, increased levels of cytokinin in plants is temporally correlated with HcPR10 expression patterns. Although the expression of cytokinin biosynthesis genes validated was not upregulated, cytokinin-related genes including chloroplast-related genes, cytokinin metabolism and cytokinin responses genes and flowering-related genes were significantly upregulated in the transgenic Arabidopsis compared to the wild type by transcriptome deep sequencing. Analysis of the crystal structure of HcPR10 revealed a *trans*-zeatin riboside (a type of cytokinin) located deep in its cavity, with a conserved conformation and protein–ligand interactions, supporting HcRP10 acts as a cytokinin reservoir. Moreover, HcPR10 in *Halostachys caspica* predominantly accumulated in vascular tissue, the site of long-distance translocation of plant hormones. Collectively, we draw that HcPR10 as a cytokinin reservoir induces cytokinin-related signal transduction in plants, thereby promoting plant growth and development. These findings could provide intriguing insights into the role of HcPR10 proteins in phytohormone regulation in plants and advance our understanding of cytokinin-mediated plant development and could facilitate the breeding of transgenic crops with earlier mature, higher yielding agronomic traits.

## Introduction

Pathogenesis-related proteins (PRs) are produced by plants in response to various abiotic and biotic stresses. Pathogenesis-related class 10 (PR-10) is a large family of intracellular PRs with more than 100 members (Xu et al., 2014). Some PR-10 genes are constitutively expressed in almost all plant parts, including vegetative and reproductive tissue, suggesting important roles in plant growth and development (Liu and Ekramoddoullah, 2006). For example, *OsPR10* in rice (*Oryza sativa*) is strongly expressed during all stages of root and flower development (Kim et al., 2008). Other PR-10 genes are induced in response to pathogens and parasites (viruses, bacteria, fungi, and insect herbivores) and to different environmental cues, indicating that they play protective roles in plants (Agarwal and Agarwal, 2014). In addition, the expression of some PR-10 genes is induced by various abiotic stresses, including osmotic, salinity, low and high temperatures, heavy metals, wounding, and ultraviolet (UV) exposure, suggesting they are important for tolerance to multiple stresses (Agarwal and Agarwal, 2014).

PR-10 proteins are multifunctional and highly conserved in three dimensional structures. Most PR-10 proteins characterized to date are small (155–163 amino acids) acidic proteins that primarily localize in both nucleus and cytoplasm (Guo et al., 2022) and have two functional domains. One domain, the phosphate-binding loop (P-loop; GXGGXG), is highly conserved among nucleotide-binding proteins, and functions as a nucleotide binding site that can activate ribonuclease activity (Lebel et al., 2010). The other domain is the Bet v 1 motif, which is characteristic of proteins from the Bet v1 subfamily. This family was named after the major birch pollen allergen Bet v 1 and many family members have been shown to have RNase activity (Liu and Ekramoddoullah, 2006). The RNase activities of PR-10 proteins contribute to the hypersensitive response of plants, which involves programmed cell death and facilitates plant resistance to different diseases (Sinha et al., 2020). PR-10 proteins might also function in the degradation of the RNAs from invading pathogens, which helps limit the growth of fungal, bacterial, and viral invaders (Kim et al., 2011).While the accurate mechanism by which PR-10 proteins plays its pivotal roles remains to be elucidated, their conserved sequence motifs and the fact that PR-10 proteins are found throughout the plant kingdom suggest they play general, indispensable roles in plants.

Based on sequence similarity, PR-10 proteins can be divided into intracellular pathogenesis-related (IPR) proteins/classic PR-10 proteins, cytokinin-specific binding proteins (CSBPs), and major latex proteins (MLPs) (Aglas et al., 2020). Despite their low primary sequence similarity (17%–25%), all PR-10 proteins contain a large Y-shaped hydrophobic cavity (Fernandes et al., 2013). This hollow cavity in the molecular core is surrounded by a seven-stranded antiparallel β-sheet wrapped around a long C-terminal α-helix, which rests on a V-shaped support formed by two short helices (Chwastyk et al., 2014). This internal cavity is viewed as a broad-specificity carrier and/or a reservoir for possible small-molecule ligands and may be responsible for the binding and intracellular transport of apolar ligands (Sliwiak et al., 2018).

Slight changes in the shape and structure of this cavity would allow the binding of different plant hormones, thereby tuning their physiologic and immunologic functions, such as cytokinins, brassinosteroid analogs or abscisic acid, as well as molecules, such as flavonoids, which are related to stress response (Lebel et al., 2010). Although structural information on PR-10 proteins is substantial, understanding of their biological function remains limited. Recent studies emphasize that PR-10 proteins’ structural and functional properties cannot be only understood by their proteinogenic properties. The selective binding of a structurally diverse spectrum of hydrophobic ligands gives PR-10 the possibility to participate in adaptive mechanisms of various physiological processes by storing and transporting ligands. Cytokinins are phytohormones involved in the regulation of plant development, growth and defense mechanisms, cell division, and the deceleration of senescence (Zubini et al., 2009). Indeed, some ‘classic’ PR-10 proteins and CSBPs bind to cytokinin molecules (Pasternak et al., 2006; Fernandes et al., 2009; Kofler et al., 2012), and a series of studies has pointed to a functional link between PR-10 proteins and cytokinins. For instance, the constitutive expression of the pea (*Pisum sativum*) gene *ABSCISIC ACID-RESPONSIVE 17* (*ABR17*, also named *PR-10.4*) enhanced germination and early seedling growth in transgenic *B. napus* as well as transgenic Arabidopsis (*Arabidopsis thaliana*). The transgenic plants showed elevated endogenous cytokinin concentrations and the upregulation of several cytokinin-responsive genes (Dunfield et al., 2007; Krishnaswamy et al., 2008). An ethylene-induced rose (*Rosa hybrida*) PR-10 family gene, *RhPR10.1*, inhibits ethylene-induced flower senescence *via* increasing the cytokinin contents (Wu et al., 2017). These findings suggest that the binding of cytokinin to PR-10 proteins may be crucial for plant growth and development. However, the biological relevance of these studies has not been further explored, most studies performed to date have focused on the structure of the PR-10 and cytokinin complex or its biological activity, which means there is still a significant gap in our knowledge of structure-function relationships. Further research on the biological function of PR-10 is related to its ligand-binding activity could provide intriguing insights into the role of PR-10 proteins in phytohormone regulation in plants, and also shed light on the control of gene expression during plant growth and development. Exploring the roles of PR-10 proteins in plant growth and development would also advance our understanding of plant development and could facilitate the breeding of crops with high-quality agronomic traits.

We previously identified an upregulated sequence (expressed sequence tag, EST) from the halophyte *Halostachys caspica* under 600 mM NaCl treatment (Liu et al., 2012). In this study, we cloned the full-length sequence of the corresponding gene by SMARTTM RACE and named it *HcPR10* (GenBank accession no. KF673356.1). Transgenic Arabidopsis plants overexpressing *HcPR10* under the control of the cauliflower mosaic virus (CaMV) 35S promoter showed enhanced growth and were developmentally ahead of the wild type (WT) at the reproductive stage. Transcriptome analysis and the measurement of cytokinin contents of transgenic *HcPR10* plants, as well as analysis of the localization of HcPR10 protein in *H. caspica*, suggested that HcPR10 regulates plant growth and development in association with cytokinin. Analysis of the HcPR10 crystal structure revealed that the protein can bind to *trans*-zeatin riboside, a type of cytokinin. In conclusion, we determined that HcPR10 promotes plant growth and development is related to its cytokinin binding activity, which providing a novel mechanistic understanding of the biological roles of PR-10 family members. We suggest that *HcPR10* could be used as a candidate gene to develop earlier mature, higher yield crops in future breeding programs.

## Materials and methods

### Plant materials and growth conditions

*H.caspica* seeds were collected in the Gurbantünggüt desert of northwestern China and germinated in plastic pots containing vermiculite medium: perlite: soil (2:1:1, v:v:v) in a growth chamber at 28°C day/20°C night under a 16-h light/8-h dark photoperiod and 20–30% relative humidity. *Arabidopsis thaliana* (accession Columbia-0) seeds were sown onto half-strength Murashige and Skoog (MS) medium and grown at 22°C under a 16-h light/8-h dark photoperiod for 12 days. Seedlings were transferred to plastic pots containing vermiculite medium: perlite: soil (2:1:1, v:v:v) and grown in a growth room under standard controlled light conditions (16-h light/8-h dark photoperiod) and 60-70% relative humidity.

### Cloning and sequence analysis of HcPR10

Total RNA was extracted from 0.2 g fresh samples of *H. caspica* assimilating branches using an RNAprep pure Tissue Kit (Tiangen Biotech (Beijing) Co., Ltd). Based on the EST sequence of *Hc11c3* from the halophyte *H. capsica* (Liu et al., 2012), amplification was performed using a combination of a SMART RACE cDNA amplification kit (Clontech) and an Advantage 2 PCR enzyme system (Clontech) according to the manufacturer’s instructions. The PCR products were cloned into the pMD18-T vector (Promega, USA), and entire inserts in the positive clones were sequenced. The corresponding full-length genomic DNA was amplified and sequenced. The primers used are listed in **Supplemental Table S1**. Sequence comparisons were performed using BLAST (NCBI), and a phylogenetic tree was constructed using the neighbor joining method with the MEGA 5 program with Poisson-corrected distance with 1,000 bootstrap replicates.

### Subcellular localization and transactivation assay

The *HcPR10* gene without the termination codon was cloned into the pCAMBIA1302-35S-eGFP vector using the primers listed in **Supplemental Table S1**. The resulting clone was introduced into Agrobacterium (*Agrobacterium tumefaciens*) strain GV3101 (Ke et al., 2012). Onion (*Allium cepa*) epidermal peels were subjected to hypertonic treatment as described previously, and immersed in medium containing an Agrobacterium cell suspension harboring the above construct to expression of the GFP fusion proteins in onion epidermal cells essentially as described (Lee et al., 2008).

Matchmaker GAL4 two-hybrid system (Clontech, CA, U.S.A.) was used for the transactivation assay, the full-length coding sequence of *HcPR10* was cloned into vector pGBKT7 using an In-Fusion HD Cloning Kit (Clontech, USA). The pGBKT7-*HcPR10* construct, pGBKT7-Lam/pGADT7-T (negative control), and pGBKT7-53/pGADT7-T (positive control) were transformed into yeast strain Y2HGold using the standard protocol of the Matchmaker Gold two-hybrid system (Clontech). The transformed yeast cells were grown on the synthetic defined (SD) media SD/-Leu/-Trp, SD/-Ade/-His/-Leu/-Trp, and SD/-Ade/-His/-Leu/-Trp/X-a-Gal plates at 30°C for 3-5 days. The diameter and color of the colonies were observed and recorded. The positive control grew well not only on SD/-Leu/-Trp, SD/-Ade/-His/-Leu/-Trp medium but also on SD/-Ade/-His/-Leu/-Trp/X-a-Gal medium and displayed a-galactosidase activity. In contrast, yeast cells harboring a negative control can only grow on SD/-Leu/-Trp medium and do not exhibit a-galactosidase activity. If HcPR10 has transactivation activity, yeast growth is consistent with positive control, and vice versa.

### Immunoblot analysis

Recombinant HcPR10 protein was purified using a Ni-NTA purification kit according to the manufacturer’s instructions (Qiagen, Germany) to prepare mouse anti-HcPR10 antibodies. Proteins were isolated as described by Breiteneder et al. (1989), transferred to a nitrocellulose membrane (Pall Corporation, Washington, NY, USA), and analyzed with protein-specific antibodies. The expression of *actin* in *H.caspica* was very stable and often used for internal reference protein (Zhang et al., 2016; Zhang et al., 2022a).

### Generation of transgenic arabidopsis lines

The full-length coding sequence of *HcPR10* was amplified and cloned into the *Kpn*I and *Pst*I sites of pCAMBIA1301 under the control of the CaMV 35S promoter. The resulting plasmid, pCAMIA1301-*HcPR10*, was introduced into Agrobacterium strain GV3101. The floral dip method was used for Arabidopsis transformation (Clough and Bent, 1998).Hygromycin resistant T_0_ seedlings expressing the *HcPR10* were selected. The independent T_1_ transgenic lines were screened for Mendelian segregation ratios 3:1 (resistant:sensitive) and obtained single insert homozygous T_2_ lines. Positive T_2_ homozygous lines were selected and confirmed by RT-PCR and Immunoblot for further analysis. Each RT-PCR run was carried out for three biological replicates and each of which corresponded to three technological repeats of separate experiments. The housekeeping gene *Atactin2* was used as the internal control (Yin et al., 2021).

### Transcriptome analysis

Samples from transgenic 42-day-old Arabidopsis tissue were sent to Novogene Corporation (Shanghai, China) for RNA extraction and sequencing. The expression level of the WT was used as a control to identify DEGs between transgenic and WT plants using the criterion | FC (fold-change) | ≥2. Cluster analysis of the DEGs was performed using Cluster software and the Euclidean Distance Array formula, and the clustering results were displayed using Java TreeView (https://github.com/tanghaibao/GOatools) and KOBAS (http://kobas.cbi.pku.edu.cn/home.do) were used to identify putative biological functions and biochemical pathways for the DEGs and to identify significantly overrepresented GO terms.

### Reverse transcription quantitative PCR

Total RNA was extracted from 0.2 g fresh samples of the rosette leaves in 42-day-old WT and transgenic *Arabidopsis* (OE1, OE9), using a Total RNA Purification Kit (Norgen Biotek Corp., Thorold, Canada), and gene expression levels were detected by RT-qPCR. To normalize gene expression levels, *Atactin2* gene was used as a reference gene (Yin et al., 2021). The PCR conditions were as follows: initial denaturation step of 30 s at 95°C, 40 cycles of PCR (95°C for 5 s, 58–60°C for 30 s). The relative expression of the detected genes was calculated using the 2^-ΔΔ^Ct method. The expression levels of growth and development-responsive genes (*AtARR6*, *AtHXK1*, *AtLHCB2.4, AtARR4, AtCATA22, AtWOX2, AtIAA4, AtSAUR10, AtSAUR68, AtATL8, AtBT2, AtGRXS3, AtUGT74E1, AtFBA5, AtGASA6, AtF11I11.190, AtDIR21, AtARR21*), flowering-related genes (*AtSOC1*, *AtFT*, *AtLFY*), salt and osmotic stress-related genes (*AtROC4*, *AtGLN2*, *AtMTRF1*, *AtBHLH100*, *AtPSRPZ*, *AtCRB*), cytokinin biosynthesis genes (*AtIPT1*, *AtIPT2* and *AtIPT3*) and cytokinin degradation genes (*AtCKX2*, *AtCKX3* and *AtCKX4*) were investigated using RT-qPCR. The primers used for the RT-qPCR analysis are listed in **Supplemental Table S1**. Data represent the mean ± standard deviation of three biological replications and each replication has at least three independent rosette leaves (n ≥ 9).

### Cytokinin measurements and detached leaf senescence assay

Approximately 0.8 g fresh samples of the aerial parts of 42-day-old Arabidopsis plants were sampled as three biological replicates (groups of samples). The samples were sent to Nanjing Ruiyuan Biotechnology Co., Ltd. to identify natural cytokinins such as zeatin (Z), *trans*-zeatin riboside (tZR), the N^6^ -(Δ^2^- isopentenyl)-adenine (iP), and isopentenyl adenosine (iPA) by the isotope internal standard method. Data represent the mean ± standard deviation of three biological replications and each replication has at least ten independent rosette leaves (n ≥ 30).

Cytokinins play an important role in delaying plant senescence (Balibrea Lara et al., 2004). In order to verify the reliability of the significant increase in cytokinin levels in transgenic plants, detached leaves from 42-day-old soil-grown Arabidopsis plants were placed on filter paper soaked in distilled water and transferred to a plate. The plates with then wrapped in aluminum foil and incubated at 23°C in the dark for the indicated times. The detached leaves were photographed and their leaf senescence phenotypes observed. The total protein contents of leaves were estimated by the Bradford method (Ni et al., 2009). The chlorophyll contents were measured according to (Jones et al., 1989). Data represent the mean ± standard deviation of three biological replications and each replication has at least three independent rosette leaves (n ≥ 9). Correlations among different phenotypes were calculated by Pearson’s correlation analysis. The significance of the correlations was tested using the “co. Test” function in R.

### Immunohistochemistry analysis

For immunolocalization, assimilating branches, leaves, and roots from 3-month-old *H. caspica* were fixed in formaldehyde-acetic acid-ethanol (70% (0.5:0.5:9) FAA) fixative. The tissues were embedded in paraffin using xylene as the carrier, sectioned with a hand microtome, and transferred to a microscope slide. Purified his-HcPR10 fusion protein was used as antigen and was injected into healthy mouse to raise polyclonal antibodies. A 1:100 dilution of polyclonal anti-HcPR10 antibody in 2% (w/v) skim milk powder (resuspend in PBST) was used as the primary antiserum and was incubated with the deparaffinized sections for 2 h at 37°C. The slides were rinsed five times with PBST for 10 min each and incubated in a 1:500 dilution of HRP-conjugated goat anti-mouse IgG antibody (Beijing Kangwei Century Biotechnology Co., LTD) for 1 h at 37°C. The samples were rinsed three times in PBST (10 min each time) and observed by a confocal microscope (TCS-MP single-photon imaging system, Leica Microsystems, Wetzlar, Germany).

### Crystallization and structure resolution

The full-length coding sequence of *HcPR10* was cloned into the 6xhistidine-MBP-tagged pRSFDuet-1 vector. The target protein was produced in *E. coli* strain BL21 (DE3) cells, which were shaken at 37°C tp OD600 (optical density at 600 nm) reached ~1.0. After cooling to 20°C for ~1 h, 0.2 mM IPTG was added to the cells to induce protein production overnight. The cells were collected by centrifugation at 5,000 g for 10 min at room temperature. The cell pellets were resuspended in initial buffer (20 mM Tris-HCl pH 8.0, 500 mM NaCl, and 20 mM imidazole) and sonicated for 5 min. The supernatant was collected by centrifugation at 25,000 g at 4°C for 1 h. The supernatant was loaded onto a nickel-charged HiTrap Chelating FF column (GE Healthcare), and the target protein was eluted using initial buffer containing 300 mM imidazole. The eluted target protein was dialyzed against initial buffer containing added His-tagged TEV protease to cleave the His-MBP tag at 4°C overnight. The dialyzed solution was reloaded onto a nickel-charged chelating column to remove both the histidine-tagged MBP and TEV protease. The flow-through was collected and further purified through a HiLoad 200 16/600 gel filtration column (GE Healthcare) with buffer containing 20 mM Tris-HCl at pH 8.0, 100 mM NaCl, and 2 mM DTT. The concentration of the purified target protein was adjusted to approximately 20 mg/mL and stored at –80°C.

Crystallization was carried out using the hanging-drop, vapor-diffusion method by mixing equal volumes of protein and well solution. The apo-form crystals were grown in a solution containing 0.1 M HEPES sodium, pH 7.5 and 1.4 M sodium citrate tribasic dihydrate at 20°C. To obtain ligand-bound HcPR10 crystals, apo-form HcPR10 crystals were soaked in crystallization buffer containing 2 mM trans-zeatin riboside. The crystals were flash-frozen in crystallization buffer containing 25% (v/v) glycerol as the cryo-protectant.

Datasets for the apo-form and ligand-bound crystals were collected at the beamlines BL17B1 and BL19U1 of the Shanghai Synchrotron Radiation Facility in China, respectively. The datasets were processed using the HKL2000 package (Otwinowski and Minor, 1997). The structure of apo-form HcPR10 was solved by the molecular replacement method using PHENIX, using the crystal structure of Bet v 1 (PDB code: 1BV1) as the model (Gajhede et al., 1996). The initial partial model was manually rebuilt in Coot (Emsley et al., 2010) and further refined by PHENIX (Adams et al., 2010). The trans-zeatin riboside-bound HcPR10 structure was also solved by the molecular replacement method using the apo-form HcPR10 structure as the model. The initial model was rebuilt in Coot and refined by PHENIX for several cycles.

### Statistical analysis

GraphPad Prism 8.0 software was used for data analysis. Significant differences were determined by Student’s test. The significance levels were *P <*0.05 (*), *P*<0.01 (**) and *P*<0.001 (***).

## Results

### Cloning and expression analysis of HcPR10; localization of HcPR10

**Figure 1:**
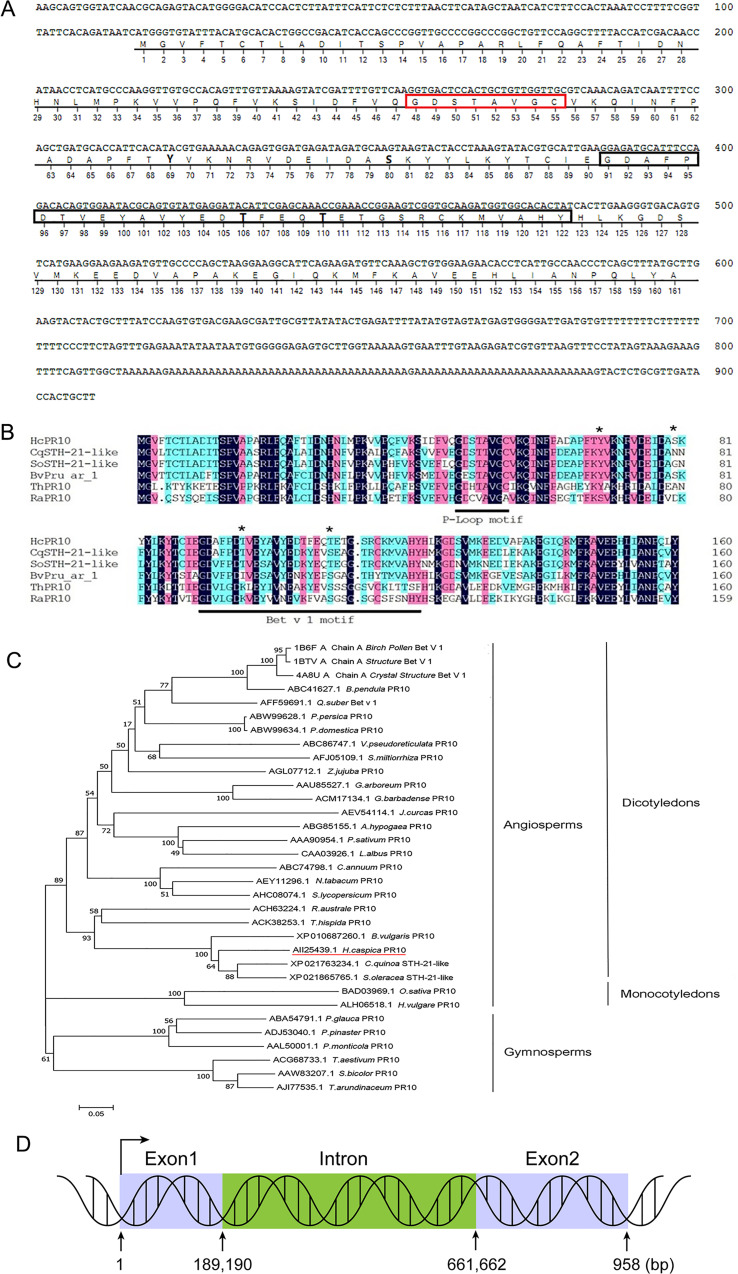
Structural and Sequence Analysis of HcPR10. **(A)** Analysis of the *HcPR10* cDNA and conserved amino acids in HcPR10. P-loop domain was framed in yellow, Bet v 1 motif was framed in gray and the putative phosphorylation sites in HcPR10 are shown in bold. **(B)** Amino acid sequence alignment of HcPR10 from *H. caspica* and related proteins from other plants. The conserved P-loop and Bet v 1 domain are marked by red and black rectangles respectively, and the four phosphorylation sites are marked by bold. **(C)** Phylogenetic tree of PR-10 proteins from different species. The GenBank accession numbers for the PR-10 sequences used for phylogenetic analyses are AII25439.1 (*Halostachys caspica*), XP_021763234.1 (*Chenopodium quinoa*), XP_021865765.1 (*Spinacia oleracea*), XP_010687260.1 (*Beta vulgaris*), ACK38253.1 (Tamarix hispida), ACH63224.1 (*Rheum austral*), ACH63224.1 (*Rheum australe*), AJI77535.1 (*Saccharum arundinaceum*), AAA90954.1 (*Pisum sativum*), ACM17134.1 (*Gossypium barbadense*), AEY11296.1 (*Nicotiana tabacum*), ABW99634.1 (*Prunus domestica*), ABW99628.1 (*Prunus persica*), ABC74798.1 (*Capsicum annuum*), ALH06518.1 (*Hordeum vulgare*), ABC86747.1 (*Vitis pseudoreticulata*), CAA03926.1 (*Lupinus albus*), AAL50001.1 (*Pinus monticola*), AAW83207.1 (*Sorghum bicolor*), AGL07712.1 (*Ziziphus jujuba*), ACG68733.1 (*Triticum aestivum*), AFJ05109.1 (*Salvia miltiorrhiza*), AHC08074.1 (*Solanum lycopersicum*), ABC41627.1 (*Betula pendula*), BAD03969.1 (*Oryza sativa Japonica Group*), ABG85155.1 (*Arachis hypogaea*), AEV54114.1 (*Jatropha curcas*), AFF59691.1 (*Quercus suber*), AAU85527.1 (*Gossypium arboreum*), ABA54791.1 (*Picea glauca*), ADJ53040.1 (*Pinus pinaster*), 4A8U_A (*Betula pendula*), 1BTV_A (*Betula pendula*), 1B6F_A (*Betula pendula*). **(D)** Diagram of the *HcPR10* gene, which consists of two exons and one intron. The blue rectangles indicate exons, and green rectangles indicate introns.

The full-length *HcPR10* cDNA sequence is 909 bp long and contains a 486-bp open reading frame encoding a polypeptide of 161 amino acids. Sequence analysis suggested that HcPR10 contains a conserved ‘P-loop’ (phosphate-binding loop) motif at residues 48-55, a Bet v 1 domain at residues 91-122, and four potential phosphorylation sites (Tyr-69, Ser-80, Thr-106, and Thr-110) (**Figure 1A**). Sequence analysis using DNAMAN software indicated that HcPR10 shares high sequence similarity with other ‘classic’ PR-10 proteins (**Figure 1B**). To clarify the relationship between HcPR10 and these proteins, we performed a phylogenetic analysis. HcPR10 belongs that the same subcluster as BvPR10 from sugar beet (*Beta vulgaris*); both belong to the ‘classic’ PR-10 proteins subfamily (**Figure 1C**). The *HcPR10* genomic clone contains one intron (190-661 bp) and two exons (1–189 and 662–958 bp; **Figure 1D**).

**Figure 2:**
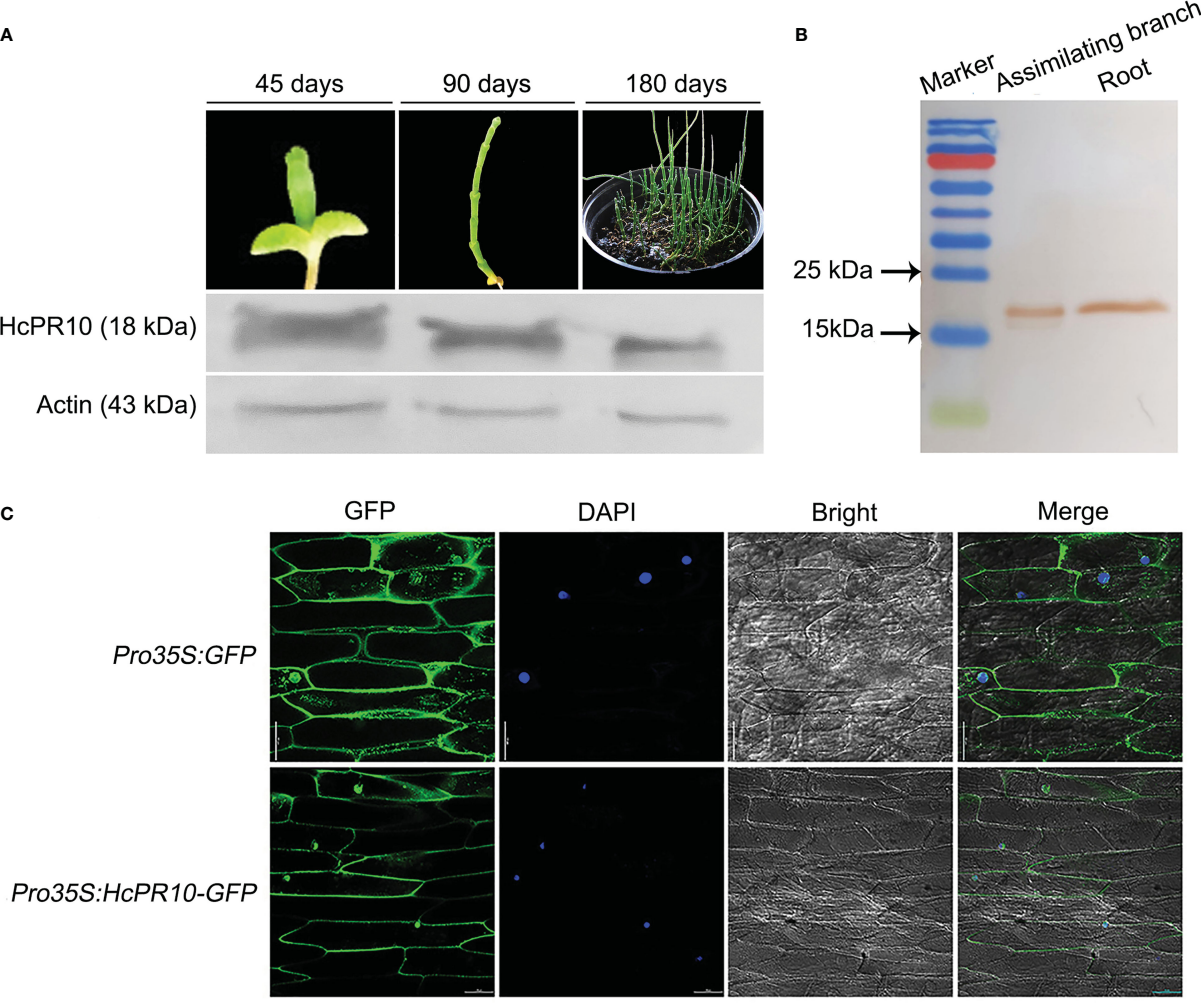
Abundance and localization of HcPR10. **(A)** HcPR10 accumulation in *H. caspica* at various stages of development (45, 90 and 180 days), as revealed by immunoblot analysis. **(B)** Confirmation of HcPR10 accumulation in assimilating branches and roots of 3-month-old *H.capsica* by immunoblot analysis. **(C)** HcPR10 localizes to the nucleus and cytoplasm, as revealed by transient expression of the *HcPR10-GFP* fusion construct in onion epidermal cells. GFP, green fluorescence; DAPI, nuclear localization; bright, brightfield; merged views were obtained by confocal laser microscopy. Scale bars, 100 μm.

HcPR10 is present in *H. caspica* at different developmental stages (**Figure 2A**) and in different tissues (**Figure 2B**), as revealed by immunoblot analysis, suggesting that HcPR10 is constitutively expressed in plants. To clarify the localization of HcPR10 in cells, we transiently expressed the *Pro35S:HcPR10-GFP* and *Pro35S:GFP* (positive control expressing only the green fluorescent protein [GFP]) plasmids in onion epidermal cells by Agrobacterium (*Agrobacterium tumefaciens*)-mediated transfection. When transformed with the GFP plasmid, green fluorescent signals were distributed throughout the cell. By contrast, we exclusively detected fluorescence in the nucleus and cytoplasm of cells expressing the fusion construct (**Figure 2C**). These results indicate that HcPR10 localizes to these intracellular regions. We detected no transactivation of the HcPR10 protein *via* a yeast two-hybrid assay (**Supplemental Figure 1**).

### Heterologous expression of HcPR10 promotes growth and development in transgenic arabidopsis

To characterize the function of HcPR10, we transformed WT Arabidopsis with a plant expression vector containing full-length *HcPR10* driven by the CaMV35S promoter. We obtained seven independent homozygous transgenic lines through repeated hygromycin selection. RT-PCR confirmed that *HcPR10* is expressed in all transgenic lines (**Supplemental Figure 2A**). We then examined HcPR10 protein levels in three transgenic lines (OE1, OE9, and OE18) and the WT by immunoblot analysis (**Supplemental Figure 2B**); all lines tested accumulated HcPR10.

**Figure 3:**
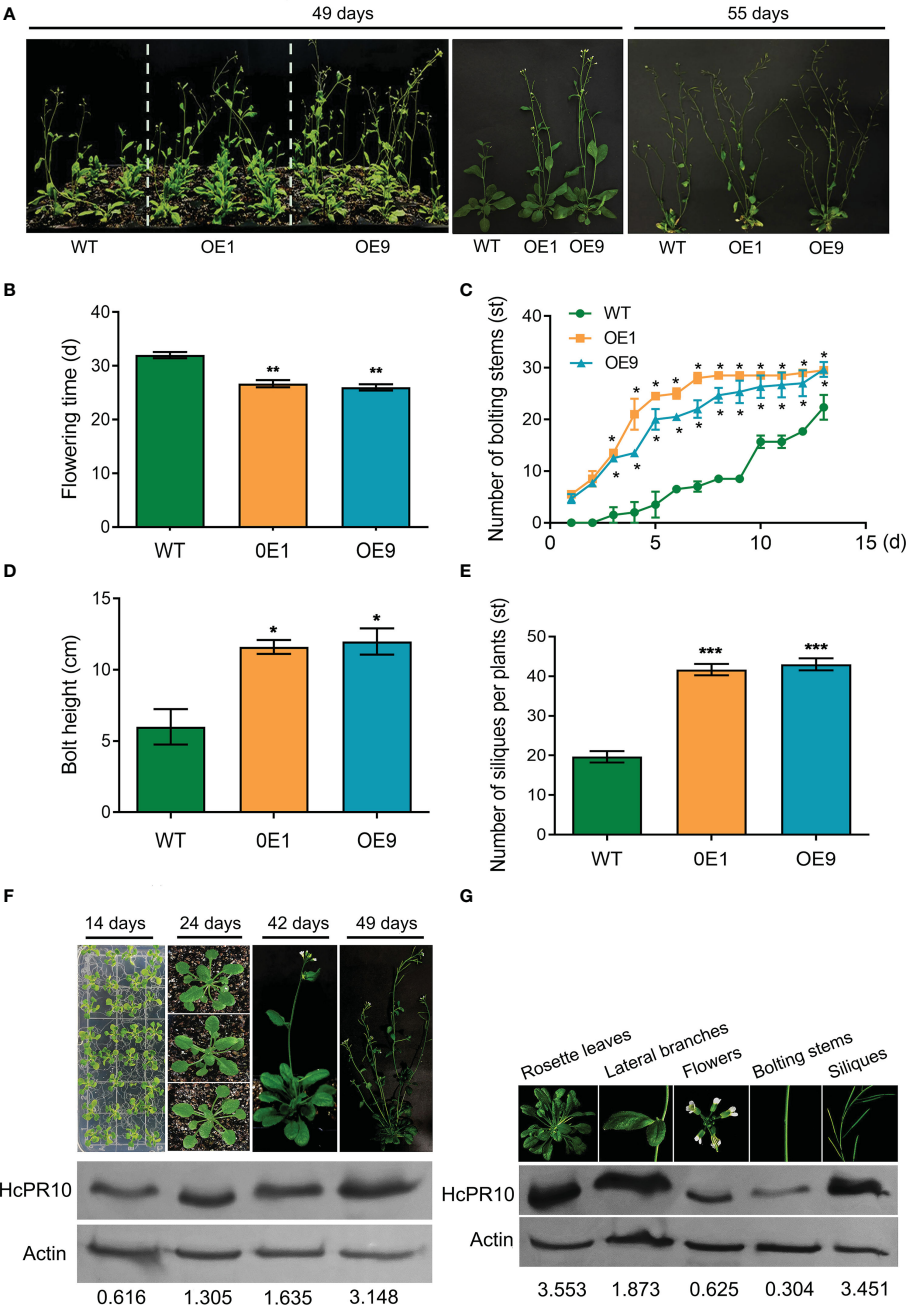
Effects of Heterologous Expression of *HcPR10* on Arabidopsis Growth and Development at the Reproductive Stage. Growth phenotypes **(A)**, flowering time; **(B)**, number of bolting stems; **(C)**, bolt height (8 days after recording the number of bolting stems); **(D)**, and number of siliques per plant **(E)** of WT, OE1 and OE9 plants. HcPR10 protein levels at different stages of growth and development **(F)**. HcPR10 protein levels in different tissues of 49-day-old transgenic plants **(G)**. Data are means ± SE using 96 plants per replicates. Significant difference in these lines was observed compared to WT plants under the same treatment. Student’s *t*-test was used: *P <0.05, **P <0.01 and ***P <0.001.

We grew transgenic (OE1, OE9) and WT plants on half-strength Murashige and Skoog (MS) medium at 22°C under controlled conditions (16-h light/8-h dark photoperiod) to explore the phenotypic changes at various stages of growth. There were no significant differences in morphology or growth characteristics between the transgenic plants and WT at the seedling or vegetative stages (*P >*0.05; **Supplemental Figure 3**). However, the transgenic lines were more developmentally advanced than the WT (**Figures 3A–E**). Time (days) to opening of the first flower was also earlier (7 days) in the transgenic lines than the WT (*P <*0.01; **Figure 3B**). The transgenic plants produced more bolting stems than the WT (*P <*0.05; **Figure 3C**) and reached a greater bolting height (*P <*0.05; **Figure 3D**). In addition, approximately twice as many siliques per plant and the number of bolting stems were present in these plants compared to WT plants (*P <*0.05 and *P <*0.001; **Figures 3C, E**).

We then examined whether the enhanced growth and development of the transgenic lines was due to the altered accumulation of HcPR10. Immunoblot analysis showed that HcPR10 accumulates in the transgenic lines at all stages of growth and development, with 2.6-fold and 5-fold higher levels in 42-day-old and 49-day-old transgenic plants vs 14-day-old plants, respectively (**Figure 3F**). In 49-day-old transgenic plants, HcPR10 protein levels were higher in rosette leaves, lateral branches, and siliques but lower in flowers and bolts compared to the WT (**Figure 3G**). These results suggest that HcPR10 positively regulates growth and development and the vegetative-to-reproductive phase transition in transgenic Arabidopsis.

### Gene expression profiling by transcriptome deep sequencing

To further explore the role of HcPR10 in promoting plant growth and development, we performed RNA-seq to investigate differences in transcript levels between 42-day-old plants of transgenic line OE9 and WT. Three biological replicates were performed per genotype. Pearson’s correlation analysis of the transcriptome data between biological replicates confirmed the generally high reproducibility of our RNA-seq datasets, as indicated by the high correlation coefficients (**Supplemental Figure 4A**). To validate the gene expression profiles obtained by RNA-seq, we examined the transcript levels of 16 cytokinin-related genes by reverse transcription quantitative PCR (RT-qPCR). The results were mainly consistent with the expression profiles obtained by RNA-seq, thus verifying the reliability of the RNA-seq data (**Supplemental Figure 4B**).

**Figure 4:**
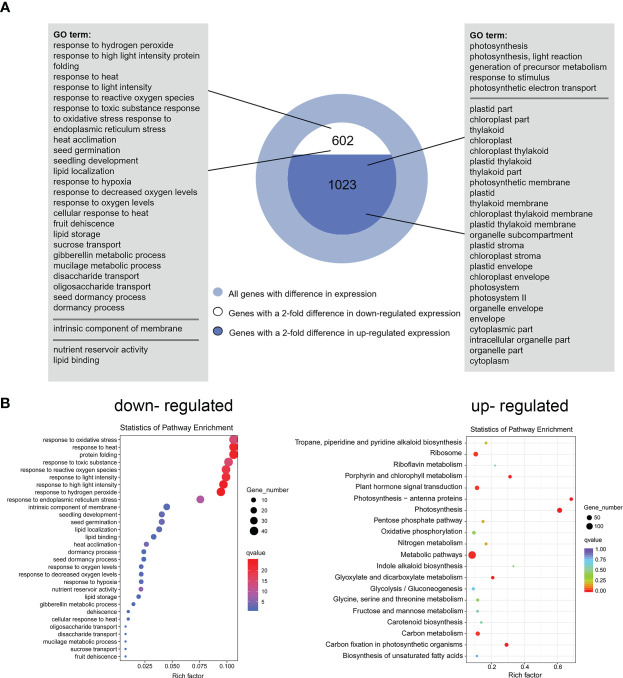
Functional Annotation of Differential Genes between Wild-Type and Transgenic *HcPR10* Arabidopsis. **(A)** Functional annotation of differential genes based on Gene Ontology (GO) analysis. **(B)** KEGG pathway analysis of differential genes.

**Figure 5:**
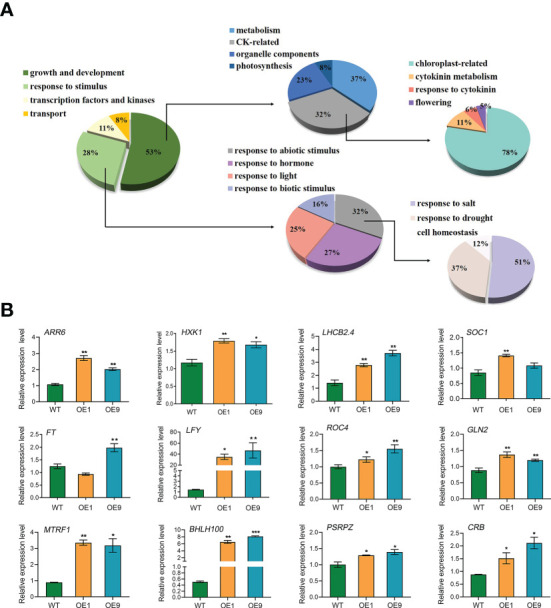
Analysis of the Upregulated Differentially Expressed Genes. **(A)** Pie charts showing the subclassification of the categories of upregulated differentially expressed genes. **(B)** Gene expression analysis of classical growth and development-related genes and abiotic stress-related genes in *HcPR10* transgenic Arabidopsis. Data are means ± SE from three independent experiments. Significant differences compared to WT plants under the same treatment using Student’s *t*-test *P <0.05, **P <0.01 and ***P <0.001.

DEGs were identified based on the following threshold values: | FC (fold-change) | ≥2 and *P* ≤0.05. 602 downregulated genes and 1023 upregulated genes were identified in the OE9 compared with WT (**Figure 4**; **Supplemental Table 2**). Gene Ontology (GO) terms indicated that these 1,023 upregulated DEGs are highly enriched in the biological processes ‘photosynthesis’, ‘light reaction’, ‘generation of precursor metabolism’ and ‘photosynthetic electron transport’. 602 downregulated DEGs are highly enriched in the biological processes ‘response to stimulus’, ‘seed germination’ and ‘lipid storage’ (**Figure 4A**). Moreover, Kyoto encyclopedia of genes and genomes (KEGG) pathway analysis revealed that these 1,023 upregulated DEGs are highly enriched in ‘Metabolic pathways’, ‘Photosynthesis’, ‘Ribosome’, ‘Plant hormone signal transduction’ and ‘Porphyrin and chlorophyll metabolism’, and 602 downregulated DEGs are highly enriched in ‘response to stress’ (**Figure 4B**). Considering advanced growth and development of the transgenic plants, we concentrated on analyzing the up-regulated differentially expressed genes. Of 1023 upregulated genes, 53% (515 genes) were involved in growth and development, including cytokinin metabolism and response, organelle components, and photosynthesis (particularly a set of chloroplast-related genes). In addition, 28% (269 genes) of upregulated DEGs were involved in responses to stimuli, mainly abiotic stimuli such as salt and osmotic. Finally, 11% (157 genes) of upregulated DEGs were enriched for the GO term transcription factors and kinases, and the final 8% (117 genes) of upregulated DEGs were enriched for the GO term transport (**Figure 5A**; **Supplemental Table 3**). Meanwhile, we analysed some of upregulated genes including flowering (*SOC1*, *FT*, *LFY)*, cytokinin-related (*ARR6*, *HXK1*, *LHCB2.4*), and salt (*ROC4*, *GLN2*, *MTRF1*) and osmotic (*BHLH100*, *PSRPZ*, *CRB*) stress by RT-qPCR, which were significantly induced in *HcPR10* transgenic plants at the reproductive stage compared to WT (*P <*0.05, *P <*0.01 and *P <*0.001; **Figure 5B**), suggesting that HcPR10 may be involved in regulating cytokinin signal transduction.

### Heterologous expression of HcPR10 increases endogenous cytokinin concentrations

**Figure 6:**
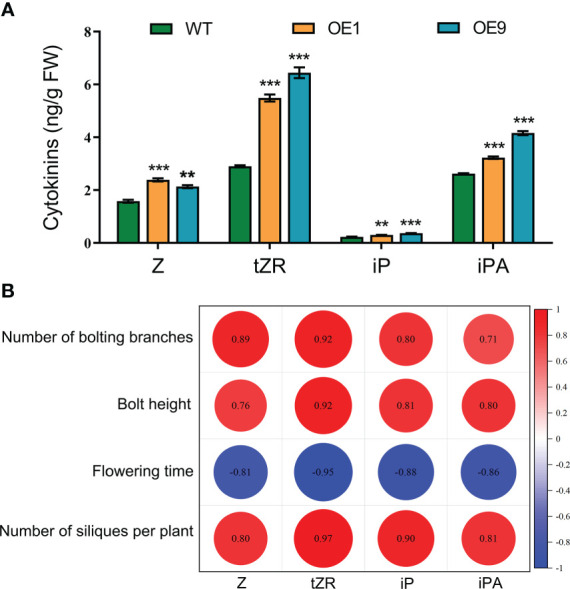
Cytokinin Contents and Pearson’s Correlation Coefficients with Agronomic Traits Related to Growth and Development in *HcPR10* transgenic plants. **(A)** Endogenous cytokinin contents in WT and transgenic plants. **(B)** Pearson correlation matrix between the contents of different types of cytokinins and key parameters related to growth and development at the reproductive stage. Horizontal and vertical directions correspond to the contents of different types of cytokinins and agronomic traits of the transgenic lines at the reproductive stage. Correlations were considered highly significant for Pearson’s product-moment correlation coefficients |r| > 0.8. Significant negative and positive correlation coefficients are indicated in blue and red, respectively. In **(A)**, data are means ± SE using 3 plants per replicates. Significant differences were determined compared to WT plants under the same treatment using Student’s *t*-test *P <0.05, **P <0.01 and ***P <0.001. Z, *cis*-zeatin; tZR, *trans*-zeatin riboside; iP, N^6^ -(Δ^2^- isopentenyl)-adenine; iPA, Isopentenyl adenosine.

To determine whether cytokinins are involved in HcPR10-regulated growth and development, we measured the endogenous levels of various types of cytokinins and their nucleoside and nucleotide precursors in 42-day-old WT and *HcPR10*-transgenic Arabidopsis plants. Two transgenic lines (OE1 and OE9) showed increased endogenous Z, tZR, iP, and iPA concentrations compared to the WT. The concentrations of Z, iP and iPA were approximately 1.2-1.6-fold higher in the transgenic plants than the WT. Importantly, the endogenous levels of tZR increased significantly (by ~2-fold) in the transgenic plants relative to the WT (*P <*0.001; **Figure 6A**). These results indicate that the heterologous expression of HcPR10 increases cytokinin concentrations in transgenic Arabidopsis.

To identify the possible functional relationships between cytokinin levels and HcPR10, we calculated the Pearson’s correlation coefficients of various cytokinin contents and the agronomic traits of the transgenic lines at the reproductive stage, representing key parameters directly or indirectly involved in growth and development. Following a Shapiro-Wilk test (Jureckova and Picek, 2007), the variables used for the Pearson coefficient calculation followed a normal distribution. The number of bolting stems, bolting height, number of siliques per plant, and the levels of four types of cytokinins were all positively correlated, whereas flowering time was negatively correlated with the levels of the four cytokinin types (*P <*0.001; **Figure 6B**).

Cytokinins delay plant senescence (Lara et al., 2004). To confirm the effect of changes in cytokinin contents in the transgenic plants, we exposed the detached aerial parts of WT and transgenic (OE1, OE9) plants to dark conditions to induce senescence. After 30 h and 60 h of dark treatment, the leaves of WT plants showed more severe chlorosis and yellowing than the leaves of the transgenic plants (**Supplemental Figure 5A**). The chlorophyll and protein contents of all plants decreased in the dark, but the transgenic plants showed higher chlorophyll and protein levels than WT plants after this treatment (*P <*0.01 and *P <*0.001; **Supplemental Figures 5B, C)**, suggesting that the transgenic plants exhibit delayed dark-induced senescence. To explore the reason for the increased cytokinin levels, we measured the expression levels of cytokinin biosynthesis and degradation genes in all plants by RT-qPCR. Genes related to cytokinin biosynthesis (*ISOPENTENYLTRANSFERASE 1* [*AtIPT1*], *AtIPT2* and *AtIPT3*) exhibited similar expression patterns in both transgenic and WT plants. However, the expression levels of genes related to cytokinin degradation (*CYTOKININ OXIDASE/DEHYDROGENASE 2* [*AtCKX2*], *AtCKX3* and *AtCKX4*) were dramatically lower in transgenic Arabidopsis relative to WT (*P <*0.001; **Supplemental Figure 6**).

### Immunoblotting and localization of HcPR10

**Figure 7:**
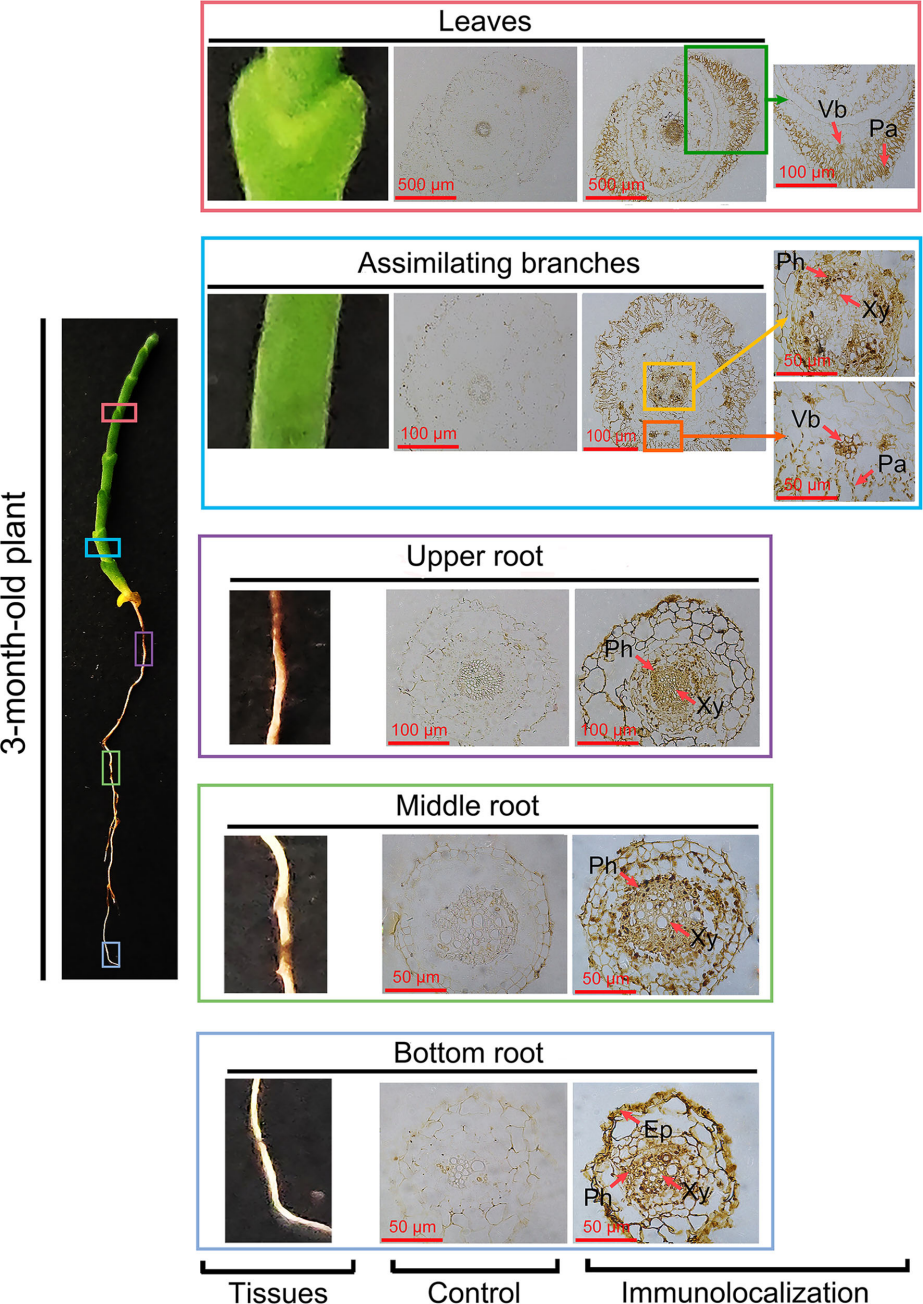
Immunolocalization of HcPR10 in *H. caspica.* For immunohistochemical analysis, sections from vegetative tissues (assimilating branches, leaves and roots) obtained with a microtome were incubated in purified anti-HcPR10 antibody (1:100 dilution). Sections were treated with pre-immune serum as a negative control. Brown staining indicates the accumulation of HcPR10. Arrows indicate specific tissues. The sections were incubated in HRP-conjugated goat anti-mouse secondary antibody (1:500 dilution) for 1 h and observed by confocal microscopy. Ep, Epidermis; Ph, Phloem; Xy, Xylem; Vb, Vascular bundle; Pa, Palisade tissue.

To investigate the role of HcPR10 in the cytokinin signaling pathway, we performed immunohistochemical analysis of assimilating branches, leaves, and roots of 3-month-old *H. caspica* with the specific anti-HcRR10 antibody generated in this study. The assimilating branches refer to the tender and succulent current-year branches (phylloclades) that take over the function of gas exchange from the degenerated leaves, thus minimizing evaporative water loss (Huang et al., 2003). We detected HcPR10 in the phloem, xylem, and palisade tissue of assimilating branches and leaves. We also detected HcPR10 in the exodermis and inner parts of the endodermis in roots, including the phloem/xylem. No immunostaining signal was observed in control sections incubated with pre-immune serum (**Figure 7**).

### Crystal structures of HcPR10 and HcPR10 in complex with trans-zeatin riboside

**Figure 8:**
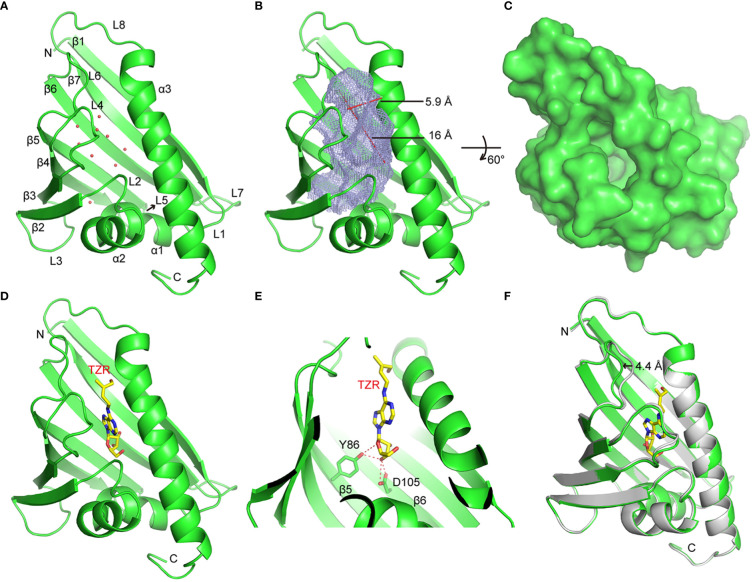
Crystal Structures of HcPR10 and HcPR10 in Complex with *Trans*-zeatin Riboside. **(A)** Cartoon representation of the apo-form HcPR10 structure, with secondary structural elements labeled. Water molecules are shown as red dots. **(B)** Mesh representation of the inner cavity of the apo-form HcPR10. **(C)** Surface representation of the top view of the apo-form HcPR10. **(D)** Structure of HcPR10 with a bound *trans*-zeatin riboside (tZR). **(E)** Details of the interaction between the tZR ligand and HcPR10. **(F)** Superimposed structures of the apo-form and ligand-bound HcPR10. The apo-form structure is colored in gray, and the ligand-bound structure is colored as shown in **(D)**.

To explore the molecular basis of HcPR10 activity, we crystalized full-length HcPR10 that had been produced in *E. coli* and solved the structure of its apo-form at 1.9-Å resolution (PDB accession code: 8H3I; **Supplemental Table 4**). Like the structures of other PR-10 proteins (Aglas et al., 2020; Morris et al., 2021), HcPR10 was composed of a seven-stranded β-sheet (β1-β7), two closely connected V-shaped α-helices (α1-α2) between β1 and β2, a long C-terminal α-helix (α3) and eight loops (L1-L8) connecting these structural elements (**Figure 8A**). HcPR10 formed a pinecone-shaped structure, with the twisted seven-stranded β-sheet and the long C-terminal helix forming the top surface of this structure and the curved V-shaped helices forming the bottom (**Figure 8A**). Inside the pinecone-shaped structure was a large cavity mainly composed of hydrophobic residues that may favor hydrophobic ligand binding. The cavity also contained several hydrophilic residues, allowing water molecules to bind inside the cavity in the apo-form structure (**Figures 8A, B**). The cavity had a volume of ~1,339 Å^3^ and contained one entrance located at the surface close to the top of the pinecone-shaped structure (**Figures 8A, B**). The entrance was surrounded by the long C-terminal helix, loops connecting α2, and β2 (L2), β3 and β4 (L4) and β5 and β6 (L6). The entrance was approximately 5.9 Å wide and 16 Å long, which was large enough for the internal insertion of a small ligand (**Figures 8B, C**).

Several PR-10 proteins were shown to bind to cytokinins (Aglas et al., 2020; Morris et al., 2021). To determine whether HcPR10 alsobinds to cytokinins, we soaked the HcPR10 crystal with *trans*-zeatin riboside (tZR) and were able to solve the structure of HcPR10 complexed with tZR at 1.75-Å resolution (PDB accession code: 8H3J; **Supplemental Table 4**). In this complex, one tZR molecule was inserted into the hydrophobic cavity of HcPR10 (**Figure 8D**). Several water molecules were also observed in the cavity. tZR adopted a linear configuration, with the ribose head inserted deep inside the cavity and the hydroxyl group of the zeatin tail positioned close to the entrance of the cavity (**Figure 8D**). Specific recognition through hydrogen bonding is mainly mediated by the ribose part of the ligand. The hydroxyl group connected to the C3 position of the ribose ring (numbered C13 in this molecule) formed a pair of hydrogen bonds with the side chain of Asp-105 and a hydrogen bond with the side chain of Tyr-86. The side chain of Tyr-86 formed another hydrogen bond with the oxygen atom of the ribose ring (**Figure 8E**). These hydrogen bonds likely facilitate the correct positioning of the ligand in the cavity. The hydrophobic environment may also help stabilize the adenine ring and the aliphatic part of the zeatin tail in the cavity.

Compared to the apo-form structure, most structural elements of ligand-bound HcPR10 superimposed well with their apo-form counterparts, except around the entrance of the cavity, where we observed conformational changes in the loops connecting β3 and β4 (L4) and β5 and β6 (L6), both of which shifted outwards to widen the entrance (**Figure 8F**). The L6 loop exhibited the most noticeable conformational change, with the tip of the loop shifted outwards by approximately 4.4 Å. These conformational changes might facilitate ligand binding and the fitting of the ligand into the cavity.

## Discussion

The PR-10 proteins comprise a unique class of highly conserved proteins and they play more different roles in plant development and defense mechanisms (Agarwal and Agarwal, 2014; Aglas et al., 2020). *HcPR10*, a *PR-10* family member cloned from *H.capsica*, was abundantly expressed at different developmental stages and in different tissues (**Figures 2A, B**), suggesting a role in plant growth and development. To explore how this gene contributed to plant growth and development, we produced transgenic Arabidopsis plants overexpressing *HcPR10*. We observed no visible differences between WT and transgenic lines at the seedling or vegetative stage (*P >*0.05; **Supplemental Figure 3**). However, the transgenic plants flowered 7 days earlier than the WT (*P <*0.01; **Figure 3B**), and produced approximately twice as many bolting stems and siliques per plant as WT plants at the reproductive stage (*P <*0.05 and *P <*0.001; **Figures 3C–E**). Notably, HcPR10 abundance in the transgenic plants was higher at the reproductive stage than at other developmental stages (**Figure 3F**). Cytokinin treatment of leaves during vegetative growth did not modify any of the evaluated plant growth variables (Leite et al., 2003), but treatment with higher levels of cytokinins resulted in an earlier transition from the juvenile to adult phase (Werner et al., 2021b). It is possible that there may be a threshold of cytokinin concentration to mediate the plant developmental transition. In the current study, transgenic Arabidopsis did not exhibit any phenotypic changes at the seedling or vegetative stage under normal conditions, perhaps due to the relatively low levels of HcPR10 accumulation compared to the reproductive stage (**Figure 3F**). We also compared HcPR10 protein levels in different tissues at the reproductive stage (**Figure 3G**). Although *HcPR10* was driven by the 35S promoter, we detected some differences in HcPR10 accumulation among tissues, which was similar to the results reported by Chakravarthi et al. (2016).

To identify the molecular mechanism behind HcPR10, we performed RNA-seq of transgenic and WT plants. Many genes involved in growth and development were differentially expressed between *HcPR10*-overexpressing and WT plants. In particular, the transgenic plants had higher transcript levels of flowering-related genes, genes involved in cytokinin metabolism and cytokinin responses, and chloroplast-related genes when compared to WT plants (**Figure 5**). Further analysis of DEGs in *HcPR10*-overexpressing plants relative to WT revealed that 22 genes of upregulated DEGs were involved in flowering regulation, some of which were cytokinin regulation genes, such as *SMALL AUXIN UPREGULATEDs* (*SAURs*), *GATA*, *CONSTANS* (*CO*), *SUPPRESSOR OF OVEREXPRESSION OF CO 1* (*SOC1*), and *FLOWERING LOCUS T* (*FT*) (**Figure 5A**; **Supplemental Table 3**). Cytokinins are a class of phytohormones that regulates plant growth, physiological activities, and yield (Li et al., 2021). Phenotypic alterations caused by changes in the expression of cytokinin-related genes appeared to be mediated by cytokinins either indirectly or directly. For example, overexpressing *SAUR*s in Arabidopsis altered the growth of hypocotyls and floral organs (Chae et al., 2012). Exogenous cytokinin application induced flowering by acting on the shoot apical meristem in *SOC1*-overexpressing transgenic Arabidopsis plants to induce mitotic activation during the transition between the two growth stages (D'aloia et al., 2011; Richter et al., 2013). CO acts as a network hub to integrate various external, internal signals into the photoperiodic flowering pathway and regulate the expression of downstream genes including *FT*, *SOC1*, and *LFY*. These three genes integrate signals from multiple flowering pathways and their expression levels eventually determine the exact flowering time (Shim et al., 2017; Yang et al., 2022). Overexpressing the cytokinin-regulated transcription factor gene *OsGATA6* in rice resulted in increased grain number (Zhang et al., 2022b).

Furthermore, some chlorophyll-related genes such as *GNC* (*GATA, NITRATE-INDUCIBLE, CARBON-METABOLISM INVOLVED*), *CGA1* (*CYTOKININ-RESPONSIVE GATA FACTOR 1*), and *LHCB6* (*LIGHT HARVESTING COMPLEX PHOTOSYSTEM II SUBUNIT 6*) involved in photosynthetic and the photosynthetic electron transport chain identified by RNA-seq were upregulated (**Figure 5A**; **Supplemental Table 3**), which seems chlorophyll plays an important role in the phenotypical changes observed in Arabidopsis transgenic plants. Moreover, the chlorophyll contents of the transgenic plants were higher than those of WT plants under normal conditions at the reproductive stage (*P <*0.01; **Supplemental Figure 5B**). Interstingly, these genes were also related to cytokinin. GNC and CGA1 are master regulators of chloroplast biogenesis that act downstream of cytokinin to mediate the development of chloroplasts from proplastids and enhance chloroplast growth and division in specific tissues (Chiang et al., 2012). *LHCB6* is a primary target of cytokinin signaling, and cytokinin directly regulates the expression of genes involved in the etioplast-to-chloroplast transition (Cortleven et al., 2016). The altered expression of these chloroplast-related genes in *HcPR10*-overexpressing plants suggested that HcPR10 helps maintain photosynthesis by regulating cytokinin levels. However, this hypothesis must be further evaluated, as gene regulation does not necessarily indicate functional relevance. Nonetheless, given the key roles of cytokinins in shoot branching, heading, and developmental transitions such as flowering and in regulating chloroplast development (Werner et al., 2021b), the growth phenotypes of transgenic *HcPR10* plants at the reproductive stage are likely related to changes in cytokinin homeostasis.

Indeed, *HcPR10*-overexpression led to significantly increased endogenous levels of several cytokinins (*P <*0.001; **Figure 6A**). The darkness-induced delayed senescence of these plants further confirmed their high cytokinin levels (**Supplemental Figure 5**), providing direct evidence that the expression of *HcPR10* in Arabidopsis modulated cytokinin levels. IP-type and tZ-type cytokinins are generally considered to be the most active natural cytokinins, whereas Z-type cytokinin generally has less activity (Petschow, 1978). Cytokinins were shown to mediate the enhanced germination and early seedling growth of Arabidopsis transgenic plants overexpressing pea *PR-10.4* (Srivastava et al., 2007). Here we demonstrated that HcPR10-mediated phenotypes including earlier flowering and increased branch number and siliques per plant correlated with increased cytokinin levels (*P <*0.001; **Figure 6B**), reflecting the roles of cytokinins in regulating vegetative-to-reproductive phase transitions (Werner et al., 2021a). In addition, the level of the highly active tZR-type form of cytokinin was 2.05-fold higher in the transgenic plants than the WT, and the levels of Z-type, iP-type, and iPA-type cytokinins increased approximately 1.2-1.6-fold in these lines (*P <*0.001; **Figure 6A**). The changes in the phenotypes of the transgenic plants were highly correlated with the levels of these four types of cytokinins, especially tZR-type cytokinin (*P <*0.001; **Figure 6B**), pointing to the prominent roles of tZR-type cytokinins in these processes. These observations support that HcPR10 increases cytokinin contents to fine-tune cytokinin-regulated gene expression, thereby enabling earlier development.

During cytokinin metabolism, the enzyme IPT is responsible for cytokinin biosynthesis, whereas CKXs irreversibly degrade cytokinins (Liu et al., 2020). Nonetheless, we observed dramatic increases in the concentrations of four types of cytokinins (*P <*0.001; **Figure 6A**), whereas *IPT1*, *IPT2* and *IPT3* expression was not upregulated in transgenic Arabidopsis (**Supplemental Figure 6**). This result suggested that the HcPR10-mediated increase in cytokinin levels was independent of the *de novo* biosynthesis pathway. Steady-state levels of active cytokinins in planta are also affected by the rate of cytokinin degradation. *CKX* genes were expressed at significantly lower levels in *HcPR10*-overexpressing compared to WT plants (**Supplemental Figure 6**) (Werner et al., 2006). The increased cytokinin level in transgenic-*HcPR10* Arabidopsis may be related to the expression of genes encoding CKX-related enzymes. However, the specific molecular mechanism of HcPR10 protein regulating cytokinin level remains unclear. We need further research to explore.

The three-dimensional (3D) structures of PR-10 proteins have been extensively studied through X-ray crystallography and/or solution nuclear magnetic resonance (NMR) spectroscopy (Pasternak et al., 2005; Pasternak et al., 2006). In-depth structural studies of the apo and ligand-bound forms of certain PR-10 proteins have provided detailed molecular insights into ligand binding (Pasternak et al., 2006; Fernandes et al., 2008; Fernandes et al., 2009). Because the molecular mechanisms triggered by the binding of PR-10 to cytokinins are poorly understood, we explored this issue by examining the crystal structure of HcPR10 alone or soaked in a cytokinin solution. We determined that recombinant HcPR10 took on the structure of a typical PR-10 protein, with a pinecone-like shape and a large hydrophobic cavity in the middle (**Figures 8A, B**).

HcPR10 was able to hold at least one tZR molecule in its central hydrophobic cavity (**Figure 8D**). Previous studies showed that PR-10 proteins can hold 2 to 3 tZ molecules in their central cavities, although the binding positions within these cavities are not conserved, suggesting that PR-10 proteins may store tZ ligands in an unspecific manner, and act as cytokinin reservoir (Fernandes et al., 2008; Fernandes et al., 2009). In the current study, we only detected one tZR molecule in the central cavity of HcPR10 (**Figure 8D**). Sequence-specific recognition of the Z moiety of the tZR molecule was not observed, which is similar to previous observations (Fernandes et al., 2008).This confirmed the cytokinin binding properties of HcPR10 from *H. capsica in vitro*, supporting its capacity of cytokinin reservoir. The riboside moiety of the tZR molecule formed several hydrogen bonds with residues in the inner side of the HcPR10 cavity, indicating that this moiety provides the tZR molecule with additional specificity, which helps correctly position the tZR molecule in the cavity (**Figure 8E**). A comparison of the apo-form structure of HcPR10 with its ligand-bound structure revealed that conformational changes mainly occurred at the entrance region of the central cavity. These changes might be required in order for the tZR molecule to plug into the entrance of the cavity (**Figure 8F**).

Although we do not know whether HcPR10 can bind other cytokinins or ligands, both the residues within the central cavity and at the entrance likely play roles in ligand selection. Since ligand binding is a dynamic process, and the side chains pointing towards the vast volume of the cavity must have some conformational freedom, one might expect PR-10 proteins to be general rather than specific ligand binders (Pasternak et al., 2006). Therefore, PR-10-cytokinin interactions might not be very strong but are unusually versatile and, indeed, when characterized by relatively low binding constants, could guarantee efficient release of the small signaling molecules (cytokinins in this case) to their final receptors (Fernandes et al., 2009).

In plants, cytokinins undergo intercellular movement and long-distance translocation from the sites of their biosynthesis to their target cells. The long-distance translocation of cytokinins is mediated by the xylem and phloem. The xylem transports substances in an acropetal manner *via* transpiration flow, and the phloem transports cytokinins downward through symplastic connections and regulates vascular patterning (Kudo et al., 2010). The transport of cytokinins across the plasma membrane primarily occurs *via* the proton-coupled multiphasic cytokinin transport systems at the plasma membrane (Cedzich et al., 2008), which function in cytokinin import, cytokinin export, or bidirectional cytokinin trafficking. In the current study, we detected high levels of HcPR10 in the xylem and phloem of *H. caspica* (**Figure 7**). The results of HcPR10 immunoblot showed that two cross-reaction bands with expected size of about 18 kDa appeared in the assimilation branches of 45- and 90-old *H.caspica* (**Figure 2A**), which may indeed trigger cross-reaction in plants. However, we only detected single and specific hybrid protein band (**Figures 3F, G**) in transgenic Arabidopsis, showing that antiserum obtained is mainly for HcPR10 protein. Therefore, the result of immunolocalization mainly represent the expression and localization of HcPR10 in *H.caspica*, which were consistent with previous studies (Han et al., 2017). Based on these findings, we suggest that the high levels of HcPR10 in xylem and phloem might be involved in the long-distance transport of cytokinins. The structural analysis support that HcPR10 is a cytokinin-binding protein that acts as a reservoir for cytokinins and helps maintain cytokinin homeostasis, thereby guaranteeing stable transmission of cytokinin signals in plant cells.

In fact, the first step of cytokinin signal transduction is the cytokinin-induced activation of the histidine kinase activity of cytokinin receptors. The signal is subsequently transmitted to the nucleus through a series of phosphorylation reactions, thus altering the transcription of genes in the nucleus (Wulfetange et al., 2011; Lomin et al., 2018). This signal transduction involves plasma membrane-associated and potential intracellular cytokinin receptors, mobile phospho-transfer proteins that shuttle between the cytoplasm and nucleus, and type-B response regulators, which are transcription factors that reside in the nucleus (Steklov et al., 2013). HcPR10 showed no transactivation potential and localized to the cytoplasm and nucleus (**Supplemental Figure 1**, **Figure 2C**), which appears to favor the cell-to-cell movement of cytokinin and local signal distribution, along with the xylem and phloem. This observation raises the question whether HcPR10 might cooperate with other intracellular or nuclear proteins in the cytokinin signaling pathway. Alternatively, HcPR10 might act as an intracellular receptor of cytokinin in cells and exhibit different activities and biological functions in specific subcellular compartments, which was similar to the results reported by Gai et al. (2018). To elucidate the mechanism underlying the functional adaptation and dynamic distribution of HcPR10, the molecular details of HcPR10-mediated cytokinin release and transport should be explored.

## Conclusions

**Figure 9:**
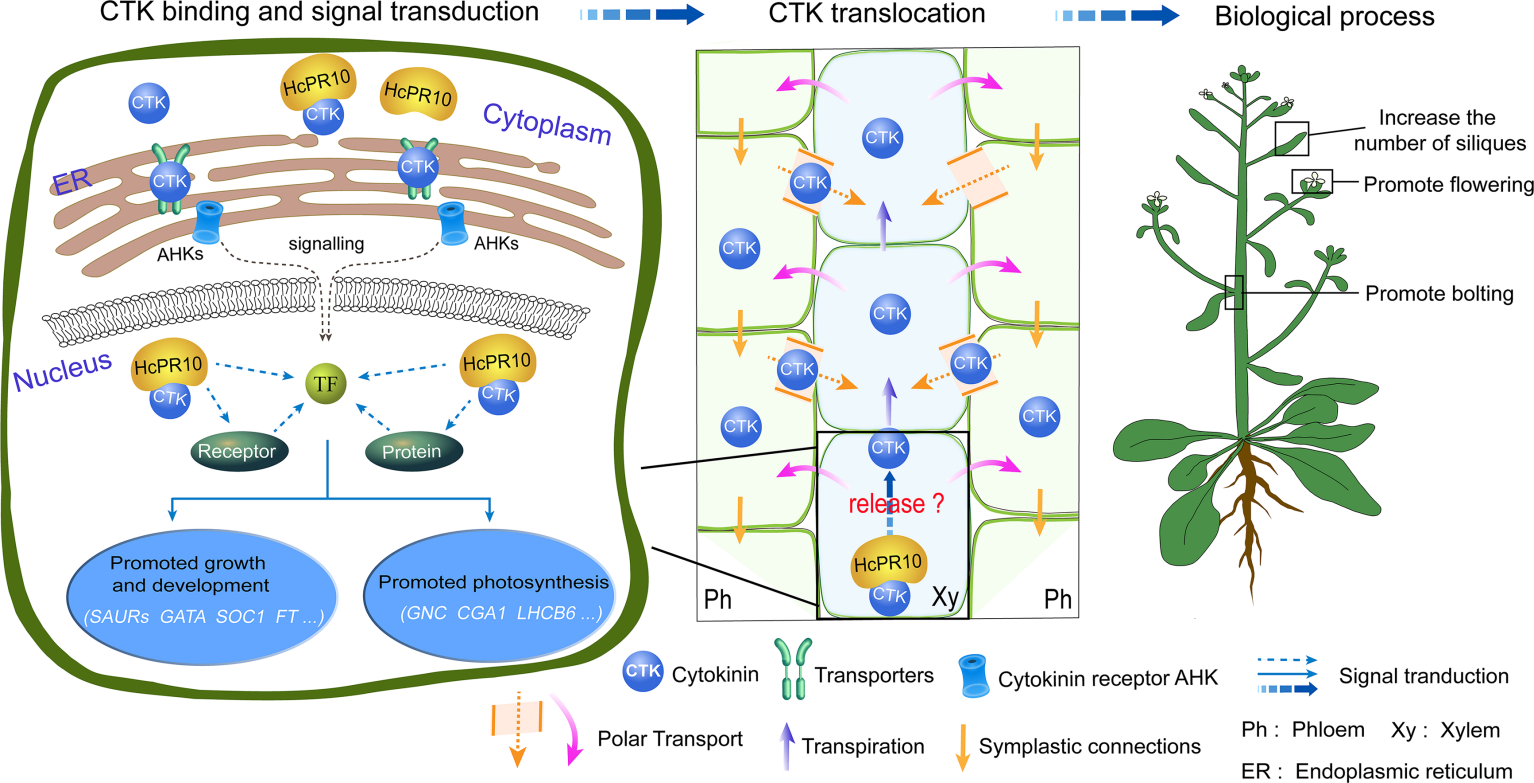
A Model for the Role of HcPR10 as a Reservoir for Cytokinin Signaling Pathways to Regulate Plant Growth and Development. The molecular/genetic cascade is shown on the left. HcPR10 with cytokinin binding activity localizes to the cytoplasm and nucleus, where is acts as a reservoir of cytokinin molecules in the aqueous environment of the plant cell and might participate in cytokinin homeostasis and signal transduction including cell metabolism, growth and development and photosynthesis. The middle panel shows the biochemical cascade. HcPR10 accumulates in the phloem and xylem, which mediate the long-distance translocation of cytokinins. The xylem is an acropetal transport system that functions *via* transpiration flow (Kudo et al., 2010). The phloem transports molecules downward through symplastic connections and regulates vascular patterning (Bishopp et al., 2011). After HcPR10 releases cytokinins, they undergo intercellular movement and long-distance translocation from the sites of their release to target cells. The localization of HcPR10 in the phloem and xylem provides an important link for intrinsic cytokinin homeostasis in plant cells that compensates for perturbed cytokinin allocation. Agricultural traits are shown to the right. cytokinin signal transduction and transport induce transcriptional reprogramming, leading to physiological changes such as promoting bolting, flowering, lateral branch and silique production.

We described a novel *PR-10* gene, *HcPR10*, from *H. caspica* and its role in grow and development. HcPR10 displays cytokinin binding activity. We propose that HcPR10 acts as a reservoir for cytokinin molecules, primarily in the xylem and phloem of *H.caspica*, where it may function in the long-distance translocation of cytokinin. Accordingly, transgenic Arabidopsis plants heterologously overexpressing *HcPR10* showed increased cytokinin contents and changes in cytokinin-related gene expression, along with enhanced growth and accelerated development compared to the WT (**Figure 9**). The discovery of the cytokinin binding ability of HcPR10 advances our understanding of the structure of this protein and uncovers a missing link in the functions of PR-10 proteins. Overall, our findings uncover the molecular basis of the effect of HcPR10 on growth and development *via* cytokinins. HcPR10 is also emerging as a potential integrator of cytokinins, which may interact with cytokinin response regulators to activate the transcriptional networks downstream of cytokinin signaling pathways, the architecture of which is not yet completely known. Future work should explore how HcPR10 releases cytokinins or interacts with other proteins to promote cytokinin signaling. As the work continues, exploring whether HcPR10 can bind to other ligands to perform other biological functions will provide more intriguing insights into the role of PR-10 proteins in phytohormone regulation in plants. Finally, HcPR10 plays distinct roles in regulating early flowering, bolting, seed production making it a promising candidate for developing high-yielding crops with excellent agronomic traits in the future. Efforts to engineer earlier mature and high yields into economically important crop plants *via* transformation of *HcPR10* are currently underway in our laboratory.

## Data availability statement

The original contributions presented in the study are publicly available. This data can be found here: Protein Data Bank, accession 8H3I and 8H3J.

## Author contributions

YW (E-mail: wangyanxju@126.com) was responsible for the conception, design, and execution of the entire experiment, as well as manuscript revision. Additionally, Yan Wang performed gene cloning and phylogenetic analysis. ZW (E-mail: wangz@bnu.edu.cn) provided technical support for analyzing the three-dimensional structure of HcPR10, contributed to the conception and design of the experiments, and revised the manuscript. YF (E-mail: 1919539784@qq.com) conducted the correlation analysis, while YR (E-mail: yanpengren@qq.com) conducted the structural analysis. Furthermore, YF and YR analyzed the data and wrote the paper. HZ (E-mail: 1758567625@qq.com) was responsible for the expression, localization, RNA-seq analysis, determination of cytokinin content, the expression of cytokinin-related genes, and senescence experiments. YH (E-mail: 1198739486@qq.com) generated transgenic Arabidopsis and conducted phenotypic observations and measurements during growth. All authors have reviewed and approved the final manuscript.
