# Supplementary material for: Halostachys caspica pathogenesis-related protein 10 acts as a cytokinin reservoir to regulate plant growth and development
Source: Front Plant Sci. 2023 Apr 26;14:1116985. doi: 10.3389/fpls.2023.1116985 (PMC10169677; doi:10.3389/fpls.2023.1116985)
Supplement: Supplementary file 2 [file DataSheet_1.pdf]

Supplementary Material

Halostachys caspica pathogenesis-related protein 10 acts as a cytokinin reservoir to regulate plant growth and development

Yu Dan Feng, Yan Peng Ren

*** Correspondence:** Yan Wang: wangyanxju@126.com, Zhan Xin Wang: wangz@bnu.edu.cn

1 Supplementary Figures and Tables

1.1 Supplementary Figures

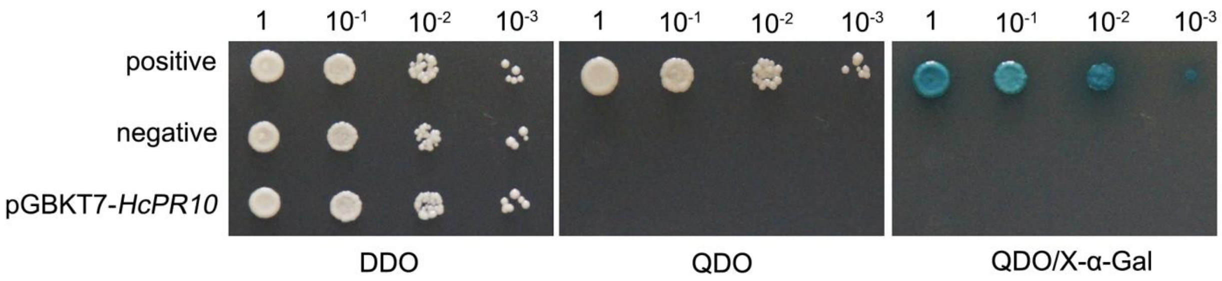

Supplementary Figure 1. Transactivation Activity Assay of HcPR10.
The transcriptional activation potential of HcPR10 was examined in a yeast two-hybrid assay. Yeast cells expressing fusion proteins were cultured, adjusted to an OD600 of 2.0, serially diluted, and 2-μL aliquots were spotted onto synthetic defined (SD) media (SD/–Leu/–Trp, SD/–Ade/–His/–Leu/–Trp and SD/–Ade/–His/–Leu/–Trp/X-a-Gal). Yeast cells harboring pGBKT7-53/pGADT7-T served as the positive control, and cells carrying pGBKT7-Lam/pGADT7-T served as the negative control. Photographs were taken after 2– 4 d of incubation at 30°C.

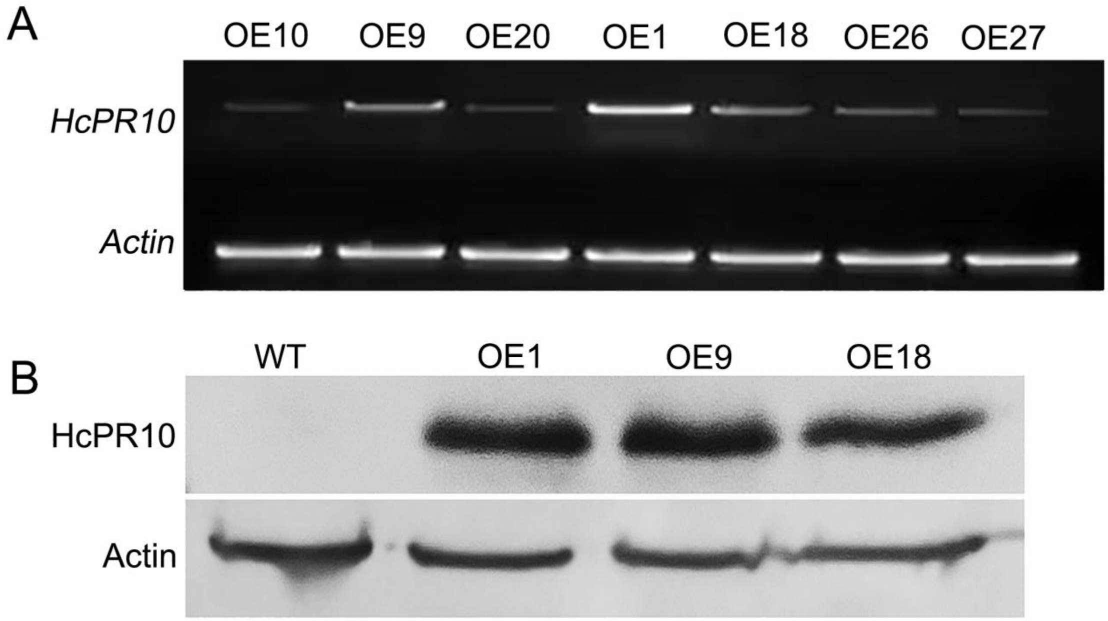

Supplementary Figure 2. Identification of Transgenic *HcPR10* Arabidopsis Plants.

(A) RT-PCR analysis of *HcPR10* expression in the seven transgenic Arabidopsis lines. **(B)** Immunoblot analysis of HcPR10 accumulation in WT and transgenic lines OE1, OE9 and OE18. *ACTIN*/ACTIN was used as the internal control for RT-PCR and immunoblotting, respectively.

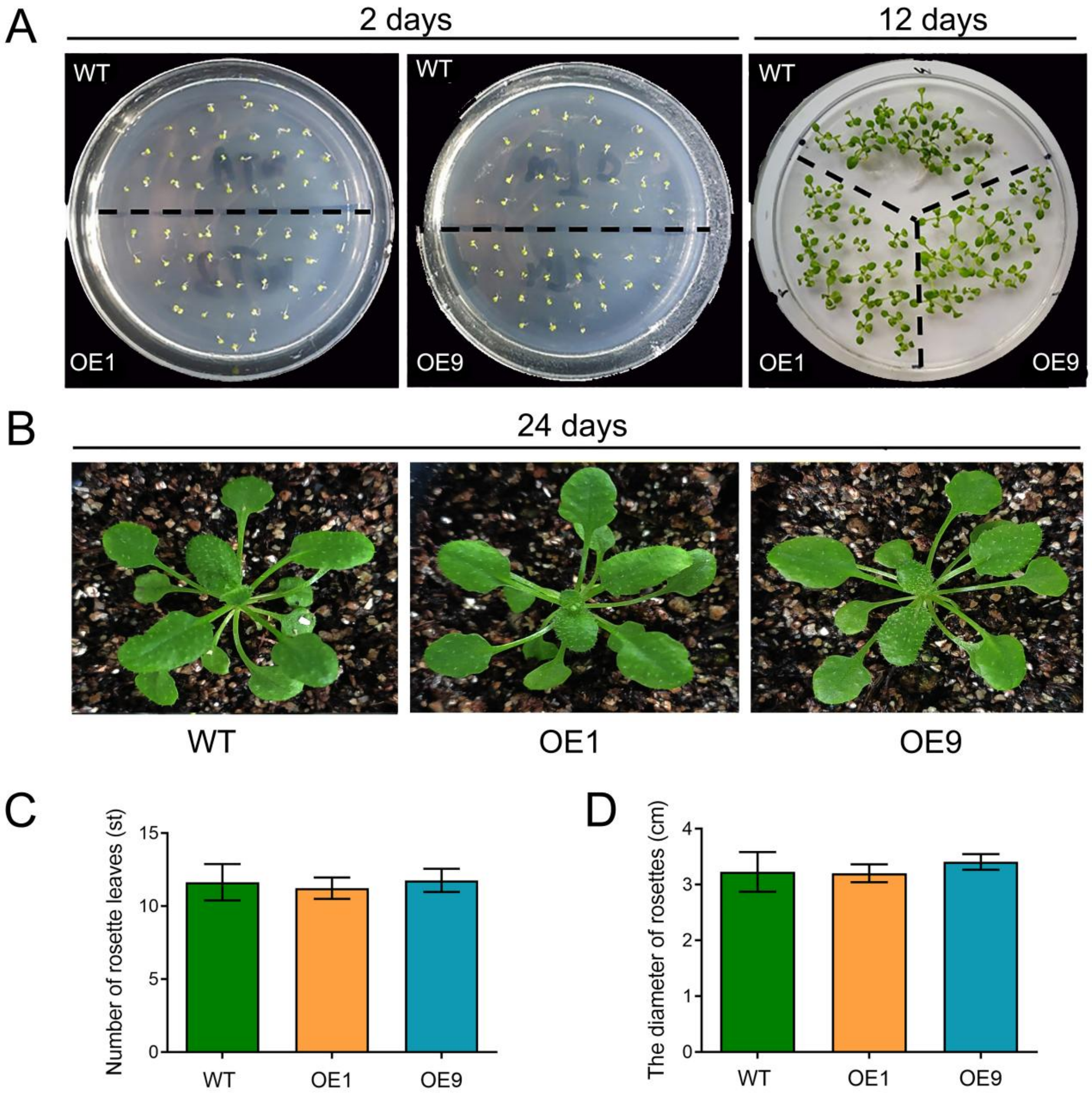

Supplementary Figure 3. Effects of Heterologous Expression of *HcPR10* on Early Growth and Development in *Arabidopsis thaliana*.

(A) Photographs of seed germination following incubation on half-strength MS medium for 2 days (left) or 12 days (right). The seedlings were grown in the greenhouse at 22°C day /18°C night, 16-h light/8-h dark photoperiod. **(B)** Phenotypes of WT, OE1, and OE9 plants after 24 days. **(C, D)** Number of rosette leaves **(C)** and rosette diameter **(D)** of 4-week-old WT, OE1, and OE9 plants. Data are means ± SE using 96 plants per replicates. No significant difference in these lines was observed compared to WT plants under the same treatment. Student's *t*-test was used: **P* > 0.05.

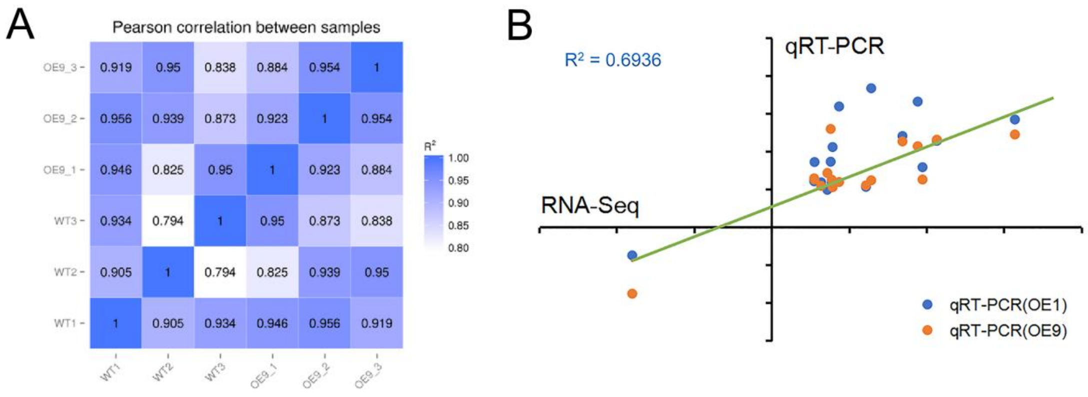

Supplementary Figure 4. Confirmation of the Reliability of the RNA-seq Data by Pearson's Correlation Analysis and RT-qPCR Profiling.

(A) Pairwise gene correlation analysis was performed by calculating the Pearson's correlation coefficient for all genes in WT and transgenic line OE9. **(B)** The correlation analysis between RNA-Seq and RT-qPCR results based on 16 randomly selected genes. Data are means ± SE from three independent experiments. Significant differences compared to WT plants under the same treatment using Student's *t*-test **P* <0.05, ***P* <0.01 and ****P* <0.001.

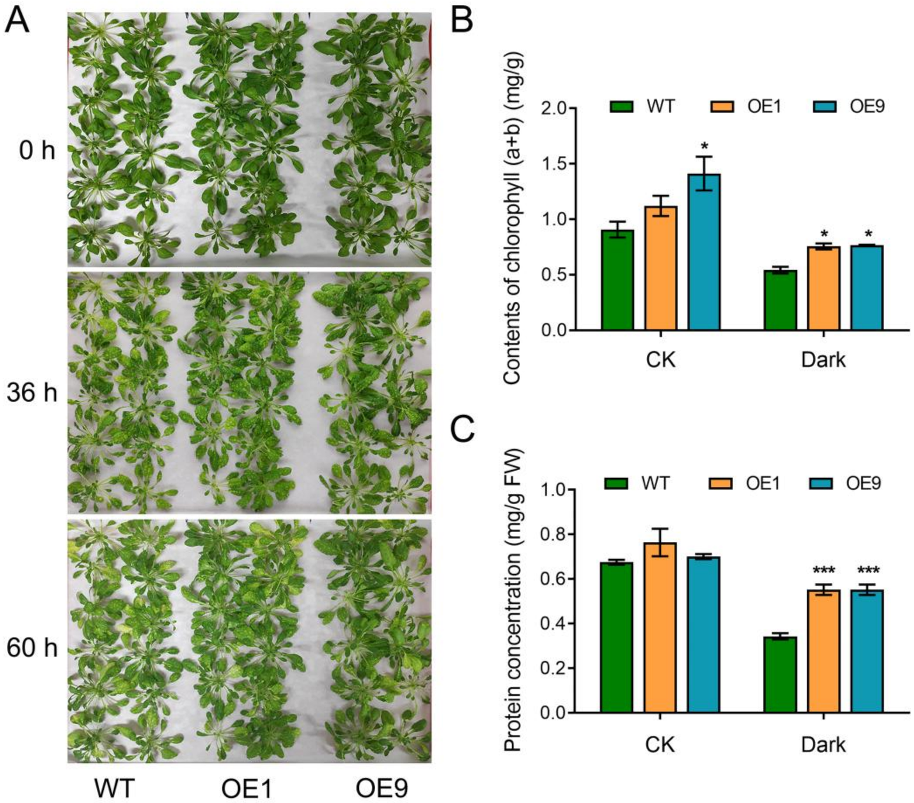

Supplementary Figure 5. Senescence is Delayed in *HcPR10* Transgenic Arabidopsis in the Dark. (A) Leaf phenotypes after 0, 36, or 60 h of dark treatment. Total chlorophyll **(B)** and protein **(C)** levels after dark treatment. CK: control, Dark: dark treatment. In (B) and (C), data are means ± SE using 3 plants per replicates. Significant differences were determined compared to WT plants under the same treatment using Student's *t*-test **P* <0.05, ***P* <0.01 and ****P* <0.001.

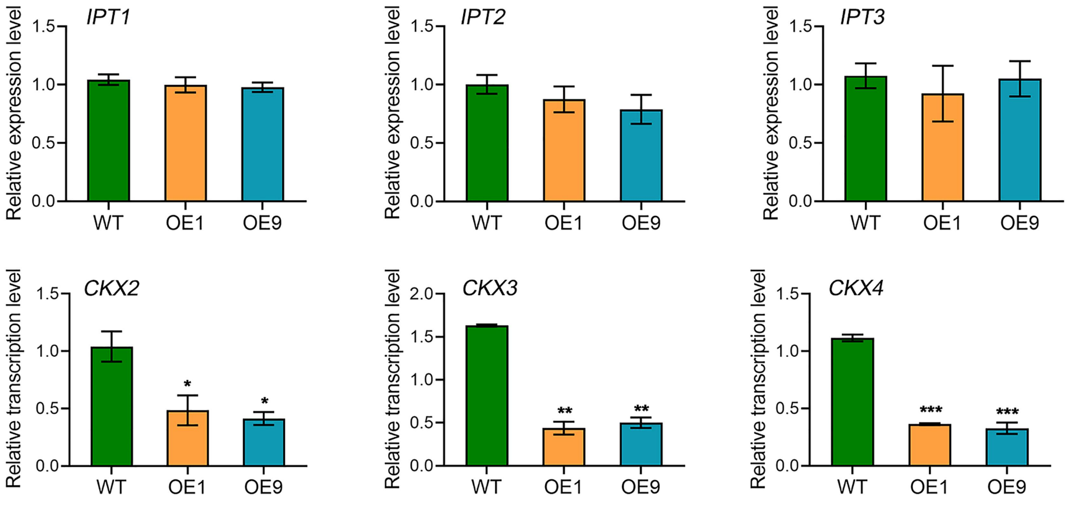

Supplementary Figure 6. HcPR10 Regulates Cytokinin-related Gene Expression.

Relative expression of cytokinin biosynthesis genes (*IPT1*, *IPT2*, *IPT3*) and cytokinin degradation genes (*CKX2*, *CKX3*, *CKX4*) in WT, OE1, and OE9 plants. Data are means ± SE from three independent experiments. Significant differences compared to WT plants under the same treatment using Student's *t*-test **P* <0.05, ***P* <0.01 and ****P* <0.001.

1.2 Supplementary Tables

Supplemental Table 1 Primers Used in This Study.

Gene	Primer	Sequence	Purpose
HcPR10	GSP1	5'-GCTGAGGGTTGGCAATGAGGTG-3'	PCR for 5′RACE
	NGSP1	5'-GTCACCCTTCAAGTGATAGTTTGCCACC-3'	PCR for 5′RACE
HcPR10	GSP2	5'-CCAGACACAGTGGAATACGCAG-3'	PCR for 3′RACE
	NGSP2	5'-GGAAGAACACCTCATTGCCAACCCTCAG-3'	PCR for 3′RACE
HcPR10	FP1	5'-CAGATAATCATGGGTGTATTTAC-3'	ORF, cDNA and genomic DNA of *HcPR10*
	FP2	5'-TCACTTTTTACCAAGCACTCTC-3'	ORF, cDNA and genomic DNA of *HcPR10*
HcPR10	EP1	5'-GCTGAGGGTTGGCAATGAGGTG-3'	flanking restriction sites
	HP2	5'-GTCACCCTTCAAGTGATAGTTTGCCACC-3'	flanking restriction sites
HcPR10	RT-S	5'-GGCTGTTCCAGGCTTTTACCAT-3'	RT-PCR
	RT-A	5'-TTTGACGCAACCAACAGCAG-3'	RT-PCR
Actin2	RT-S	5'-CCAAAGGCCAACAGAGAGAAGAT-3'	RT-qPCR
	RT-A	5'-TGAGACACACCATCACCAGAA-3'	RT-qPCR
AtIPT1	RT-S	5'-TCATGACAGAACTCAACTTCCACC-3'	RT-qPCR
	RT-A	5'-ATAAAGCTTCTAATTTTGCACCAAATGCCGC-3'	RT-qPCR
AtIPT2	RT-S	5'-CGCGGTACCGTCATGATGATGTTAAACCCTAGC-3'	RT-qPCR
	RT-A	5'-ATAGTCGACTGATATATAAATCAATTTACTTCTGC-3'	RT-qPCR
AtIPT3	RT-S	5'-CCGCCTGAAGCCGACTTAA-3'	RT-qPCR
	RT-A	5'-TTTAGGACGGATTCAATGGAGAGA-3'	RT-qPCR
AtCKX2	RT-S	5'-GAATGGTGGAATTGGTGGTC-3'	RT-qPCR
	RT-A	5'-AAGATGTCTTGCCCTGGAGA-3'	RT-qPCR
AtCKX3	RT-S	5'-CTCGGCTAAAGACGGAGTTG-3'	RT-qPCR
	RT-A	5'-TCAAAAGCCTCCCAATTGTC-3'	RT-qPCR
AtCKX4	RT-S	5'-CTCTGCGCTTCTCACGACTTCGGTA-3'	RT-qPCR
	RT-A	5'-CATAAACCCTGGAGCGAAACCTAGAG-3'	RT-qPCR
ARR4	RT-S	5'-GGTGGTGTTTCTTGTTTACGAA-3'	RT-qPCR
	RT-A	5'-ACAATACGATCAACGAGACTGT-3'	RT-qPCR
GATA22	RT-S	5'-GGAGACACGACTAAAATTGACG-3'	RT-qPCR
	RT-A	5'-CGAGTTAGTTCCTGTCATGTCT-3'	RT-qPCR
WOX2	RT-S	5'-CAGATTTGACAGATTTGCCCTC-3'	RT-qPCR
	RT-A	5'-GGAAAAAGAGGGAGTGTTTTCC-3'	RT-qPCR
IAA4	RT-S	5'-GGCTCAGACTTTGTACCTACTT-3'	RT-qPCR

	RT-A	5’-ATCCTTAGCCTCTTACAAGACG-3’	RT-qPCR
SAUR10	RT-S	5’-AAGTCGGTACATCGTTCCTATC-3’	RT-qPCR
	RT-A	5’-AGACTTCATCACAAGGAATGGT-3’	RT-qPCR
SAUR68	RT-S	5’-GTTTACACTGCGGATCAAGTAC-3’	RT-qPCR
	RT-A	5’-GATTGGTCCTTCTGTGGGAATA-3’	RT-qPCR
SAUR14	RT-S	5’-GCCAAACTGTTGTCATCATCTT-3’	RT-qPCR
	RT-A	5’-CACCCATTGGATGATCAAATCC-3’	RT-qPCR
ATL8	RT-S	5’-TTCTTCTTTGTGCACTGACATG-3’	RT-qPCR
	RT-A	5’-TCCGTTAAACAGATAGCACACT-3’	RT-qPCR
BT2	RT-S	5’-CAACAAAAAGTCAATGACTGCG-3’	RT-qPCR
	RT-A	5’-GACGTATCAAAAGCTGAAGACC-3’	RT-qPCR
GRXS3	RT-S	5’-AACTCGATGAGATTAGCAGAGG-3’	RT-qPCR
	RT-A	5’-TGAGATGGAGACTCATGACTTG-3’	RT-qPCR
UGT74E1	RT-S	5’- CATAGTTATGGTTTGAGCGGTG-3’	RT-qPCR
	RT-A	5’- AATCATTCGCATTCAGAATCGG-3’	RT-qPCR
GASA6	RT-S	5’-AAGTTATGGACCAGGAAGTCTG-3’	RT-qPCR
	RT-A	5’-CATTTAGCACAACACTTTTGGC-3’	RT-qPCR
DIR21	RT-S	5’-CTTTCAGGTTCGGAAGAGGATA-3’	RT-qPCR
	RT-A	5’-TATATTCGACCACAGCATCTCC-3’	RT-qPCR
ARR21	RT-S	5’-ATCAGTTATGGAGATCGCAGTT-3’	RT-qPCR
	RT-A	5’-TCCGATGACATAACTAAGACCG-3’	RT-qPCR
ARR6	RT-S	5’- CCTGTTCACTCGCAGCTCAA -3’	RT-qPCR
	RT-A	5’- AGCGAAAAAGGCCATAGGGG -3’	RT-qPCR
HKX1	RT-S	5’- CCAATTCCAACAAACAAACGTG -3’	RT-qPCR
	RT-A	5’-AATGAGCATCTGAAATGTCACG-3’	RT-qPCR
LHCB2.4	RT-S	5’- ATTAGCTATATGGGCGTGTCAA -3’	RT-qPCR
	RT-A	5’- TTCAACTCTGAAAACGCTTCTG -3’	RT-qPCR
SOC1	RT-S	5’-TATGGTGAGGGGCAAAACTC-3’	RT-qPCR
	RT-A	5’-TCCTATGCCTTCTCCCAAGA-3’	RT-qPCR
FT	RT-S	5’-AGAGAGGTGACTAATGGCTTGG-3’	RT-qPCR
	RT-A	5’-CAGTGGGACTTGGATTTTCGTA-3’	RT-qPCR
LFY	RT-S	5’-ACGGAGACGATTGCAAGAAGA-3’	RT-qPCR
	RT-A	5’-TACGATAAACGGATGCTCCCT-3’	RT-qPCR
ROC4	RT-S	5’- TCTATGCAGATGGTTCACACTT -3’	RT-qPCR
	RT-A	5’- CTTAATGGGTGATGCATAGTGC -3’	RT-qPCR
GLN2	RT-S	5’-CCAATTCCAACAAACAAACGTG-3’	RT-qPCR
	RT-A	5’-AATGAGCATCTGAAATGTCACG-3’	RT-qPCR
MTERF10	RT-S	5’-GAATGCGTTCAGAAGAGTGATC-3’	RT-qPCR
	RT-A	5’-GTGTTCAACACTAGACACCAAC-3’	RT-qPCR
BHLH100	RT-S	5’-TTCTATGAAACCGACGACGTAT-3’	RT-qPCR
	RT-A	5’-GTTGCCGAAACACTTAACTTCT-3’	RT-qPCR
PSRP2	RT-S	5’- GCAGCTCACTAAACTTGTTGAA -3’	RT-qPCR
	RT-A	5’- ATTGTAGCAAACCCAAATCGAC -3’	RT-qPCR

CRB	RT-S	5'-TCAAAGAGGGACATCAGGTTAC-3'	RT-qPCR
	RT-A	5'-CTGCTGAAAGACTTGACTTCAC-3'	RT-qPCR

Supplemental Table 3 Functional list of genes differentially expressed between transgenic *HcPR10* and wild-type *A.thaliana* (OE/WT).

Function	Examples of regulated genes	Gene ID	FoldChange
**Cytokinin related**			
515 genes, including 389 chloroplast-related, 23 PhotosystemII, 11 CYP450, 11 NDH,10 Photosystem I PsaA/PsaB, 5 flowering regulation, 9 GRXS, 9 LHCB, 7 UGT, 6 RPL, 5 RPL, 4 LHCA, 3 PNSB, 4 PNSL, 4 YCF, 3 ARR, 3 POR, 2 CHLI, 1 GLN,1 CGA1 genes	*CYP76C1*	AT2G45560	2.83
	PSBA	ATCG00020	9.60
	NDHA	ATCG01100	8.14
	LHCB1.1	AT1G29920	4.14
	PSAB	ATCG00340	9.29
	GRXS3	AT4G15700	12.55
	UGT76C2	AT5G05860	3.53
	RPL15	AT3G25920	2.98
	LHCA6	AT1G19150	4.57
	PNSB3	AT3G16250	3.61
	PNSL1	AT2G39470	3.66
	YCF2-B	ATCG00860	7.84
	ARR4	AT1G10470	2.87
	PORA	AT5G54190	4.56
	CHLI2	AT5G45930	3.02
	GLN2	AT5G35630	2.72
	CGA1	AT4G26150	4.86
**Abiotic stress**			
178 genes, including 4 HSP, 2 MYB, 3 NAC, 4 RBCS, 2 zinc finger C2H2, 1 ROC4 genes	*LQY1*	AT1G75690	3.91
	MYB76	AT5G07700	3.74
	NAC019	AT1G52890	3.75
	RBCS-1B	AT5G38430	4.15
	MTERF10	AT2G34620	3.74
	LEA2	AT1G02820	5.67
	ZAT10	AT1G27730	2.87
	ROC4	AT3G62030	2.99
**Biotic stress**			
91 genes, including 4 PDF and 3 WRKY genes	*PDF1.3*	AT2G26010	14.92
	WRKY54	AT2G40750	3.96
**Transcription factor and kinases**			
157 genes, including 14 BHLH, 12 ERF/AP2, 4 CCT, 3 WRKY, 3 ZAT, 5	*BHLH137*	AT5G50915	3.01
	ERF021	AT1G71450	9.30

PDF, 2 WAKL, 2 PKS, 1 WAG, 1 MAPKKK13, 1 SRK2C genes	*WRKY53*	AT4G23810	4.00
	COL2	AT3G02380	5.44
	ZAT7	AT3G46090	4.74
	PDF1.2A	AT5G44420	10.54
	WAKL8	AT1G16260	2.62
	WAG1	AT1G53700	3.29
	MAPKKK13	AT1G07150	4.94
	PKS4	AT5G04190	2.21
	SRK2C	AT1G78290	2.55
**Photosynthesis**			
127 genes, including 4 PPD, 9 LHCB,4 LHCA, 7 cytochrome genes	*PPD2*	AT2G28605	2.99
	LHCB6	AT1G15820	2.90
	LHCA4	AT3G47470	2.14
	PETB	ATCG00720	7.65
**Transport**			
117 genes, including 8ATPsynthase, 2 CPN60B, 2 GLR, 1 PPT2 genes	*PPT2*	AT3G01550	5.27
	ATPA	ATCG00120	7.16
	CPN60B4	AT1G26230	2.59
	GLR3.5	AT2G32390	4.59
**Cell wall**			
53 genes, including 5 expansin, 4 XTH, 3 PER genes	*ATEXPA1*	AT1G69530	4.77
	XTH10	AT2G14620	6.21
	PER39	AT4G11290	3.77
**Auxin action**			
46 genes, including 14 SAUR-like, 4 Aux/IAA and 3 BT genes	*SAUR68*	AT1G29490	8.52
	IAA5	AT1G15580	4.68
	BT2	AT3G48360	7.53

Supplemental Table 4 Data collection and refinement statistics.

	**HcPR10 (apo form)**	**HcPR10/ trans-zeatin riboside**
**Data collection**		
Space group	P3221	$P3_221$
Cell dimensions		
a, *b*, *c* (Å)	59.43, 59.43, 92.02	59.46, 59.46, 91.66
(°)	90.00, 90.00, 120.00	90.00, 90.00, 120.00
Resolution (Å)	50.00-1.90 (1.93-1.90)*	50.00-1.75 (1.78-1.75)
R_{merge}	0.089 (0.660)	0.121 (1.090)
$I / \sigma I$	25.8 (3.0)	35.5 (2.0)

Completeness (%)	100.0 (99.7)	100.0 (100.0)
Redundancy	19.0 (15.7)	18.9 (17.6)
**Refinement**		
Resolution (Å)	22.46-1.90	34.24-1.75
No. reflections	15377	19482
R_{work} / R_{free}	0.199/0.248	0.209/0.231
No. atoms		
Protein	1272	1272
Ligand	/	25
Water	93	101
B-factors (Å^2)		
Protein	29.9	31.0
Ligand/ion	/	42.1
Water	35.5	38.4
R.m.s. deviations		
Bond lengths (Å)	0.011	0.003
Bond angles (°)	1.097	0.673

*Values in parentheses are for highest-resolution shell.
